# Genomic, transcriptomic, and proteomic approaches towards understanding the molecular mechanisms of salt tolerance in *Frankia* strains isolated from *Casuarina* trees

**DOI:** 10.1186/s12864-017-4056-0

**Published:** 2017-08-18

**Authors:** Rediet Oshone, Mariama Ngom, Feixia Chu, Samira Mansour, Mame Ourèye Sy, Antony Champion, Louis S. Tisa

**Affiliations:** 1 Department of Molecular, Cellular and Biomedical Sciences, University of New Hampshire, 46 College Rd, Durham, NH 03824-2617 USA; 2 Laboratoire Mixte International Adaptation des Plantes et microorganismes associés aux Stress Environnementaux, Centre de Recherche de Bel-Air, Dakar, Sénégal; 3 Laboratoire Campus de Biotechnologies Végétales, Département de Végétale, Faculté des Sciences et Techniques, Université Cheikh Anta Diop, Dakar, Sénégal; 4 Laboratoire Commun de Microbiologie Institut de Recherche pour le Développement/Institut Sénégalais de Recherches Agricoles/Université Cheikh Anta Diop, Centre de Recherche de Bel-Air, Dakar, Sénégal; 5 Faculty of Science, Suez Canal University, Ismalia, Egypt; 6 UMR DIADE, Institut de Recherche pour le Développement, Montpellier, France

**Keywords:** Actinobacteria, Actinorhizal symbiosis, Comparative genomics, Salt stress, Salt tolerance, Transcriptomics

## Abstract

**Background:**

Soil salinization is a worldwide problem that is intensifying because of the effects of climate change. An effective method for the reclamation of salt-affected soils involves initiating plant succession using fast growing, nitrogen fixing actinorhizal trees such as the *Casuarina*. The salt tolerance of *Casuarina* is enhanced by the nitrogen-fixing symbiosis that they form with the actinobacterium *Frankia*. Identification and molecular characterization of salt-tolerant *Casuarina* species and associated *Frankia* is imperative for the successful utilization of *Casuarina* trees in saline soil reclamation efforts. In this study, salt-tolerant and salt-sensitive *Casuarina* associated *Frankia* strains were identified and comparative genomics, transcriptome profiling, and proteomics were employed to elucidate the molecular mechanisms of salt and osmotic stress tolerance.

**Results:**

Salt-tolerant *Frankia* strains (CcI6 and Allo2) that could withstand up to 1000 mM NaCl and a salt-sensitive *Frankia* strain (CcI3) which could withstand only up to 475 mM NaCl were identified. The remaining isolates had intermediate levels of salt tolerance with MIC values ranging from 650 mM to 750 mM. Comparative genomic analysis showed that all of the *Frankia* isolates from *Casuarina* belonged to the same species (*Frankia casuarinae*). Pangenome analysis revealed a high abundance of singletons among all *Casuarina* isolates. The two salt-tolerant strains contained 153 shared single copy genes (most of which code for hypothetical proteins) that were not found in the salt-sensitive(CcI3) and moderately salt-tolerant (CeD) strains. RNA-seq analysis of one of the two salt-tolerant strains (*Frankia sp.* strain CcI6) revealed hundreds of genes differentially expressed under salt and/or osmotic stress. Among the 153 genes, 7 and 7 were responsive to salt and osmotic stress, respectively. Proteomic profiling confirmed the transcriptome results and identified 19 and 8 salt and/or osmotic stress-responsive proteins in the salt-tolerant (CcI6) and the salt-sensitive (CcI3) strains, respectively.

**Conclusion:**

Genetic differences between salt-tolerant and salt-sensitive *Frankia* strains isolated from *Casuarina* were identified. Transcriptome and proteome profiling of a salt-tolerant strain was used to determine molecular differences correlated with differential salt-tolerance and several candidate genes were identified. Mechanisms involving transcriptional and translational regulation, cell envelop remodeling, and previously uncharacterized proteins appear to be important for salt tolerance. Physiological and mutational analyses will further shed light on the molecular mechanism of salt tolerance in *Casuarina* associated *Frankia* isolates.

**Electronic supplementary material:**

The online version of this article (doi:10.1186/s12864-017-4056-0) contains supplementary material, which is available to authorized users.

## Background

Soil salinization is a worldwide problem that is intensifying because of the effects of climate change. Globally, 20% of total cultivated and 33% of irrigated agricultural land are affected by high salinity. Salinized areas are expanding at an alarming rate of 10% per annum due to a variety of factors encompassing low precipitation, high surface evaporation, weathering of native rocks, irrigation with saline water, and poor agricultural practices [1]. By 2050, more than 50% of the arable lands are predicted to be salinized [2]. Various methods are used to reclaim salt-affected soils. One effective and commonly used method in the reclamation of salt-affected soils involves initiating plant succession using fast-growing, nitrogen-fixing actinorhizal trees such as the *Casuarina* [3]. Actinorhizal plants of the genus *Casuarina* are notable for high salt tolerance [4] and have been used as a green barrier [5–7]. Some *Casuarina* species are found growing naturally near brackish waters and swamps or in saline soils [8]. In hydroponic medium supplemented with adequate nitrogen, *Casuarina glauca, Casuarina obesa*, and *Casuarina equisetifolia* var. incana will withstand up to 500 mM NaCl [5].

Actinorhizal plants form a nitrogen-fixing symbiosis with the actinobacteria *Frankia* that results in the formation of root nodule structures where the bacteria are located. The bacteria convert atmospheric dinitrogen into a biologically useful form and supply the plant with nitrogen. Reciprocally, the plant supplies the bacteria with carbon and energy sources. *Frankia* associate with a broad range of dicotyledonous angiosperms distributed among 24 genera and 8 families [9, 10]. The symbiosis with *Frankia* allows actinorhizal host plants to colonize harsh environmental terrains including highly contaminated, dry, poorly-drained, nutrient-poor and salinized soils [11, 12].

Like their plant partners*, Frankia* strains isolated from *Casuarina* and *Allocasuarina* are more NaCl tolerant than other *Frankia* strains isolated from actinorhizal plant species not normally growing under sodic conditions [13]. *Frankia* sp. strain CcI6, isolated from the nodules of *Casuarina cunninghamiana* trees growing in Egyptian soil, is highly NaCl tolerant, exhibiting a minimum inhibitory concentration (MIC) value of 1000 mM [14]. *Frankia* sp. strain CcO1, also from *C. cunninghamiana*, showed a high tolerance level up to 500 mM NaCl [3]. Similarly, *Frankia* sp. strain Ceq1, isolated from *C. equisetifolia,* is able to withstand up to 500 mM NaCl [3, 4]. However, there is great variation in salt tolerance among the different *Casuarina* associated *Frankia* isolates, with tolerance values ranging from 100 mM to 1000 mM [3, 14]. It is of great interest to the scientific community to link the observed tolerance level to the genetic make-up of the strains. To date, more than 38 *Frankia* genomes have been sequenced and annotated, and 33 of them have been deposited to the NCBI database. The sequenced *Frankia* strains include *Casuarina* isolates from a broad range of geographic locations [15–22]. The availability of several sequenced *Casuarina* genomes provides an opportunity to combine phenotypic studies on the strains with comparative genomics and transcriptomics analyses. This study could shed light on known and novel mechanisms of salt tolerance that could help explain the observed disparity in salt tolerance among the different *Frankia* strains isolated from *Casuarina* trees.

For many microbes, there are several well-known mechanisms by which they adapt to fluctuations in osmolarity or salt including: (1) re-establishing osmotic balance by accumulating low molecular weight organic compatible solutes [23–25], (2) exclusion of Na^+^ ion from cells via the action of a Na^+^/ H^+^ antiporter and Na^+^-ATPase [24], (3) altering membrane composition through changes in fatty acid saturation or phospholipid composition to better cope with the changed turgor pressure [26], (4) reactive oxygen species scavenging to prevent the oxidative degradation of lipids, also known as lipid peroxidation [24], (5) restoration of the native folding of proteins through the actions of molecular chaperons [27].

Compatible solutes encompass a restricted range of highly water soluble, osmotically active, low molecular weight amino acids and their derivatives, sugars or sugar alcohols, other alcohols [28] and inorganic cations such as K^+^ [29]. Commonly employed compatible solutes include the sugar trehalose, the amino acids proline, serine and glutamate [30], quaternary ammonium compounds such as glycine betaine and proline betaine [31], polyamines, and organic solutes [30]. The strikingly limited number of compatible solutes used in all forms of life from bacteria to higher organisms reflect the challenge of finding solutes that are compatible with cellular functions. The osmotic function of a compatible solute depends on the degree of methylation and length of the hydrocarbon chain [32]. Evolutionary selection for a compatible solute depends on the osmotic function as well as on other secondary functions such as its contribution towards heat and cold tolerance [33]. Accumulation of compatible solutes helps to avoid external osmolality-triggered water fluxes along the osmotic gradient causing either swelling in hypotonic environments or plasmolysis under hypertonic ones. Osmotic adaptation using compatible solutes is characterized by a minimal requirement for genetic change and a high degree of flexibility in allowing organisms to adapt to wide ranges of external osmolarity [34].

Salt stress can upset the balance between different cellular processes. The uncoupling of different pathways leads to the transfer of high energy electrons to molecular oxygen (O_2_), causing formation of reactive oxygen species, ROS [35]. ROS cause oxidative damage to proteins, DNA and lipids [36]. Oxidative stress results in the oxidative degradation of lipid membranes, also referred to as lipid peroxidation. The cell needs to be equipped with a mechanism for dealing with the degradation products, including over 200 types of aldehydes, many of which are highly reactive and toxic [37].

Cells growing in high salt medium also face a loss of intracellular water, which creates an environment of low water activity and high ionic strength inside the cell. Proteins risk permanent unfolding in the resulting intracellular environment. An effective mechanism to counter this is increasing the activities of molecular chaperones which restore protein function and structure [27].

The deleterious effects of salt stress are first experienced by the cell membrane which separates the interior of the cell from the outside environment [38]. Various kinds of stresses including, but not limited to, heat shock, cold shock, osmotic shock and salinity stress cause disruption of membranes, thereby affecting membrane-linked physiological processes such as transport, enzyme activities and signal transduction. Maintaining correct fluidity of the bilayer over a wide range of salinity determines the extent of cell survival during salt stress [39]. Management of the lipid profile in response to salinity involves induction of fatty acid desaturases, which help to synthesize unsaturated fatty acids from saturated fatty acids [40]. The inherent salt tolerance of the organism dictates such responses and is a key factor accounting for the disparity in salt tolerance between organisms [41].

In this study, we attempted to understand the mechanisms of salt-tolerance in *Frankia* and have taken a comprehensive molecular and genomic approach to address this question. The salt stress tolerance levels for several *Frankia* strains isolated from *Casuarina* trees were assayed and two salt-stress tolerant strains (*Frankia* sp. strain CcI6 and Allo2) which could withstand up to 1000 mM NaCl and one relatively salt-sensitive strain (*Frankia casuarinae* strain CcI3), which could withstand only 475 mM NaCl were identified. The other *Casuarina* isolates had intermediate levels of salt-tolerance. Comparative genomics were used to link the observed difference in phenotype to the underlying genetic make-up. Transcriptome and proteome profiling of one of the two highly salt-tolerant strains was carried out under salt and osmotic stress conditions to identify genes involved in salt and osmotic stress responses.

## Methods

### *Frankia* strains and growth conditions

*Frankia* sp. strain CcI6 [42], *Frankia* sp. strain Allo2 [43], *Frankia* sp. strain Thr [44], *Frankia* sp. strain BMG5.23 [45], *Frankia* sp. CeD, *Frankia* sp. CgI82, *Frankia casuarinae* strain CcI3 [46, 47], *Frankia* sp. strain BR, *Frankia alni* strain ACN14a [46, 48], *Frankia* sp. strain EAN1pec [49], *Frankia* sp. strain DC12 [50], and *Frankia inefficax* strain EuI1c [51, 52] were used in this study. For all strains, except for *Frankia inefficax* strain EuI1c, *Frankia* sp. strain EAN1pec, *Frankia* sp. strain ACN14a, and *Frankia* sp. strain DC12, stock cultures were grown and maintained in 5 mM propionate basal growth medium supplemented with NH_4_Cl as described previously [53]. For the other strains, proportionate as a carbon source was replaced with 20 mM glucose (*F. inefficax* strain EuI1c and *Frankia* sp. strain DC12) and 20 mM succinate (*Frankia* sp. strain EAN1pec and *F. alni* strain ACN14a). Unless specified otherwise, all *Frankia* cultures were incubated at 28 °C.

### Salt sensitivity assay

The salt tolerance levels of *Frankia* strains were determined by measuring the total cellular protein content and/or total cellular dry weight after growth under salt or osmotic stress. For total cellular protein determination, a 24-well plate growth assay was used as described previously [14]. Briefly, cells were grown in propionate basal medium with or without 5 mM NH_4_Cl containing different concentrations of NaCl [0–1100 mM] or sucrose [0–1100 mM]. For strains ACN14a, EuI1c, DC12 and EAN1pec, propionate was replaced with the appropriate carbon source described above. The inoculum was adjusted to 40 μg/ml of total protein and the plates were incubated at 28 °C for 14 days. Growth was measured by total cellular protein content as described below. Growth yield was determined by subtracting the protein content of the inoculum.

For total cellular dry weight determination, *Frankia* strains were inoculated into 25 ml of basal growth medium containing different concentrations of NaCl or sucrose [0–1000 mM]. The inoculum concentration was adjusted to 40 μg/ml protein. The *Frankia* cells were grown for 14 days at 28 °C. Growth was measured by total cellular dry weight as described below. Growth yield was determined by subtracting the dry weight of the inoculum.

To evaluate the levels of tolerance, the following two parameters were used: maximum tolerable concentration (MTC) and minimum inhibitory concentration (MIC). The MTC value is the highest concentration of salt which does not affect the growth, while the MIC value is the lowest concentration of salt that inhibits growth.

### Protein content and dry weight determination

Total cellular protein content was measured by the bicinchonic acid (BCA) method [54] per the manufacturer’s specifications (Pierce, Rockford, IL, USA) and bovine serum albumin was used as a standard. Cells solubilized in 1 N NaOH were boiled at 95 °C for 10 min and centrifuged at 13,000 g for 5 min. Triplicate measurements were made for each sample. Total cellular dry weight was determined using tarred polycarbonate membranes [55].

### Vesicle induction and nitrogenase activity

To determine vesicle production and nitrogenase activity, *Frankia* cells were harvested after 14 days of growth in medium supplemented with 5.0 mM NH_4_Cl and washed three times with buffer containing 20 mM morpholinepropanesulphonic acid (MOPS) and 10 mM KH_2_PO_4_ buffer at pH 6.8. The washed cells were inoculated into growth medium lacking an external nitrogen source and containing different concentrations of NaCl or sucrose. The cultures were incubated at 30 °C for 4 days. The vesicle numbers were determined as described previously [56]. The activity of the nitrogenase enzyme was determined by the acetylene reduction assay as described previously [56].

### Accession numbers

For bioinformatics analysis, genome sequences and their annotations including amino acid and nucleotide FASTA files were obtained from the NCBI database (http://www.ncbi.nlm.nih.gov) under GenBank accession numbers [NZ_AYTZ00000000.1, NZ_LRTJ00000000.1, NZ_JENI00000000.1, NZ_JPGU00000000.1, NZ_JDWE00000000.1, NZ_JPHT00000000.1, NC_007777.1, NC_008278.1, NC_008578.1]. RNA-seq information is available in the NCBI Gene Expression Omnibus database under accession number GSE95217.

### Pan genome analysis

The web platform OrthoVenn [57] was used to identify orthologous gene clusters. OrthoVenn uses a modified version of the heuristic approach named OrthoMCL [58] to identify ortholog groups. An E-value cut off of 1e^−5^ was used for all-to-all protein similarity comparisons. An inflation value of 1.5 was used for the generation of orthologous clusters using the Markov Cluster Algorithm [59]. To determine single copy orthologs among the most tolerant, moderately tolerant and most sensitive strains, a modified Lerat method was used [60]. As a stringent criterion for homology, only gene pairs representing a bit score value equal to or higher than 30% of the maximal possible bit score value were considered homologous genes. A Venn diagram in R was used to construct the four-way Venn diagram of shared CDSs between salt-tolerant (2 strains), moderately salt-tolerant (1 strain), and the relatively salt-sensitive strain.

### Average nucleotide identity, average amino acid identity and average genomic distance

The average nucleotide identity (ANI) and average amino acid identity (AAI) between strains was estimated by using reciprocal best hits (two-way ANI or two-way AAI), as previously described [61]. Genome to genome distance was calculated using the web platform GGDC 2.1 according to the standard operating procedure previously described [62]. GGDC 2.1 BLAST+ was chosen as the alignment method for finding intergenomic matches.

### Phylogenetic analysis

A concatenated maximum parsimony phylogenetic tree was generated from 394 conserved single copy pan-orthologous genes determined by a modified Lerat method [60]. The rationale for including only single-copy genes representing species divergences was to minimize potential errors caused by gene duplication. The tree was calculated by determining the ratio of the bit score to the maximal bit score (i.e. protein match against itself). As a stringent criterion for homology, two genes are considered homologous only if the bit score value for the pair is at least 30% of the maximal bit score. The 30% cutoff maximized the number of families containing genes, and is optimal for the interspecific identification of homologous sequences [63].

### RNA-seq sample preparation and data analysis

To analyze gene expression of the salt-tolerant strain under salt stressed conditions, RNA-seq analysis was performed on one of the two salt-tolerant strains (CcI6). Cultures were grown for 7 days at 28 °C in 5 mM propionate, 5 mM NH_4_Cl basal growth medium [51] alone or supplemented with 200 mM NaCl or sucrose. The bacteria were harvested and the pellets were frozen at −80 °C until needed. Total RNA was extracted using a modified RNeasy Midi kit (Qiagen Sciences, Valencia, CA). Frozen bacterial pellets were resuspended in 0.5 mL TE buffer, pH 8, supplemented with 5 mg/ml lysozyme and incubated at room temperature for 10 min. RLT buffer (2 ml) supplemented with 1 μl/ml β-Mercaptoethanol (β-ME) was added to each sample and the pellets were homogenized. Subsequently, the RNeasy midi kit procedure was followed as per the manufacturer’s recommendation with one major modification: after addition of ethanol to the lysate, the RNeasy mini kit procedure, instead of the RNeasy midi kit procedure, was used. RNA samples were treated with DNase I (New England Biolabs, Ipswich, Massachusetts) per the manufacturer’s instructions. RNA was quantified using Qubit RNA assay (Invitrogen) and Nanodrop 2000c spectrophotometer (Thermo Scientific, Wilmington, Delaware) according to manufacturers’ specifications. The quality of each RNA sample was determined using the Agilent 2100 Bioanalyzer (Agilent, Santa Clara, CA) according to the Prokaryote Total RNA Nano protocol. RNA quality was represented by RNA integrity number (RIN value), which ranged from 1 to 10, with 10 representing the most intact RNA. Samples with RIN value greater than or equal to 8 were used for downstream analysis. Ribosomal RNA was removed from 2 to 4 μg of total RNA by the use of the MicrobeExpress kit (Ambion, Foster City, CA) according to the manufacturer’s specifications. The MEGAclear kit (Life Technologies, Carlsbad, CA) was used to remove tRNA according to manufacturer’s specifications. cDNA libraries were prepared using the TruSeq RNA Sample Prep Kit (Illumina, San Diego, CA) as described by the manufacturer. The cDNA library was verified for appropriate fragment size (approximately 250 bp) on an Agilent 2100 Bioanalyzer (Agilent, Santa Clara, CA) according to the DNA 1000 protocol described by the manufacturer. The Qubit dsDNA BR Assay (Invitrogen, Carlsbad, CA) was used to determine the cDNA concentration of each library according to manufacturer’s recommendations. Libraries were normalized to 10 nM with 10 mM Tris-HCl, pH 8.5, supplemented with 0.1% Tween 20. Illumina sequencing was carried out at Hubbard Genome Center at the University of New Hampshire on an Illumina HiSeq 2500 platform. Reads were separated on adapter assignment and pre-processed through CASAVA 1.8.3. The resulting FASTQ files of sequence reads were processed using CLC Genomics Workbench 9.0 (CLC bio, Cambridge, MD). Adapters were trimmed from reads by searching on the forward and reverse strands. The ends of reads were quality trimmed based on quality scores from a base-caller algorithm using a limit value of 0.05. The high quality trimmed reads were mapped to *Frankia* sp. strain CcI6 gene regions. Reads mapping to rRNA operons were excluded from downstream analysis. Mapping parameters were as follows: The maximum number of mismatches allowed was 2. The minimum length fraction was set so that at least 50% of the read length aligns to the reference sequence. The minimum fraction of identity between the read and the reference sequence was set at 80%. A read that matched to more than 10 distinct places in the reference was not mapped. If the read matched to multiple distinct places, but below 10 different locations, it was randomly assigned to one of the distinct places. After mapping, the expression level for each gene was tabulated in terms of the unique number of reads mapping to that gene. All RNA-seq experiments were normalized by the total number of reads. A gene was expressed if it had at least one unique sequence read aligned with it. To determine differential gene expression, statistical analysis on proportions was carried out. Two-sided *p*-values for multiple biological replicates were computed using Baggerley’s test [64].

### Proteome analysis of salt-stressed *Frankia* sp. strain CcI6 and *Frankia* sp. strain CcI3

Cultures were grown for 7 days at 28 °C in 5 mM propionate, 5 mM NH_4_Cl basal growth medium [53] alone or supplemented with 200 mM NaCl or sucrose. *Frankia* mycelium from 50 mL culture was harvested by centrifugation at 3,400×g for 20 min and resuspended in 2 mL of lysis buffer [10 mM Tris–HCl (pH 7.4), 1 mg/mL MgCl_2_, 50 μg/mL DNase, 50 μg/mL RNAse, and 50 μg/mL lysozyme]. A French pressure cell was used to lyse the cells at 137,895 kPa. Lysed samples were centrifuged at 16,200×g and the supernatant fluid containing soluble proteins was collected. Protein samples were quantified using the Bradford assay [65]. One milligram of soluble protein was precipitated with 10% (*v*/v) trichloroacetic acid (TCA) in acetone solution containing 20 mM dithiothreitol (DTT) overnight at −20 °C. The samples were centrifuged at 16,200×g for 30 min at 4 °C to pellet the proteins. Traces of TCA were removed from the pellet by washing with 20 mM DTT in acetone. The protein pellet was dissolved in 300 μL of rehydration buffer [7 M urea, 2 M thiourea, 5% (m/v) DTT, 2% (m/v) Triton X-100, 2% (*v*/v) immobiline pH gradient (IPG) buffer, 0.02% bromophenol blue]. The solubilized proteins (1 mg) were used to rehydrate an 11 cm Immobiline™ DryStrip pH gradient strip at pH 4–7 (GE Healthcare Biosciences, Pittsburgh, PA). The protein sample suspended in rehydration buffer was evenly distributed along the lanes of a strip holder and the Immobiline strips were rehydrated with the gel side facing down. DryStrip cover fluid (GE Healthcare Biosciences) was used to cover the strips to prevent evaporation and crystallization of the urea. Rehydration proceeded for 12 h. The rehydrated strips were moved to an Isoelectric focusing (IEF) tray and were positioned with the gel side facing down. Wet ProteomIQ™ IPG Wicks (Proteome Systems, Woburn, Massachusetts) were placed at the anode and the cathode to collect excess salt and other contaminants. The strips and the wicks were covered with 50 mL of DryStrip cover fluid. The IEF tray was placed in an IsoelectrIQ 2 unit (Proteome Systems, Woburn, MA) and isoelectric focusing was performed under the following settings: 100–10,000 V gradient for 8 h and 10,000 V constant for 8 h. Strips were stored gel side up at −80 °C until the second-dimension protein separation step. Strips were washed in 1× sodium dodecyl sulfate (SDS) running buffer [0.2 M glycine, 25 mM Tris-base, 0.1% (m/v) SDS] and reduction of the proteins was undertaken at room temperature by incubating the strips in SDS equilibration buffer [50 mM Tris–HCl (pH 8.8), 6 M urea, 30% (*v*/v) glycerol, 2% (m/v) SDS, trace of bromophenol blue] supplemented with 65 mM DTT for 20 min. Alkylation reaction was performed by washing the strips in deionized water followed by incubation in the same SDS equilibration buffer, supplemented with 135 mM iodoacetamide (IAA), instead of with DTT, for 20 min. The strips were rinsed in 1× SDS running buffer and loaded on a 12% SDS – Polyacrylamide gel (16 × 16 cm) with the plastic backing against one of the glass plates. Strips were sealed in place with 1% (m/v) agarose in TAE buffer (40 mM Tris, 20 mM Acetate and 1 mM EDTA, pH 8) supplemented with trace amounts of bromophenol blue for tracking purposes. Proteins were separated by electrophoresis at 100 V for 30 min followed by 200 V for 5 h. Protein spots were visualized by incubating the gels in Coomassie Blue stain [0.1% Coomassie Brilliant Blue R-250, 10% (*v*/v) glacial acetic acid, 50% (*v*/v) methanol] followed by destaining with a destain solution [10% (*v*/v) ethanol, 5% (*v*/v) glacial acetic acid]. Differentially expressed spots were excised from the gel and placed in a 0.5 mL Eppendorf tube. Gel pieces were washed three times by adding fresh 50 μL of 25 mM NH_4_HCO_3_/50% acetonitrile (ACN) each time and vortexing for 15 min. The gel pieces were incubated in 25 μL DTT solution (10 mM DTT in 25 mM NH_4_HCO_3_) at 56 °C for 1 h. The supernatant was discarded and 100 μL of IAA solution (55 mM IAA in 25 mM NH_4_HCO_3_) was added to the samples. The samples were incubated at room temperature in the dark for 45 min. The supernatant was discarded and samples were washed by adding 100 μl of 25 mM NH_4_HCO_3_ and vortexing for 10 min. The supernatant was discarded and gel pieces were dehydrated by adding 100 μl of 25 mM NH_4_HCO_3_/50% ACN solution and vortexing for 10 min. The dehydration step was repeated twice. Gel pieces were completely dried under a speed vacuum for 20 min. Five microliters of trypsin solution (40 ng/μL in 25 mM NH_4_HCO_3_) were added to the gel pieces. An additional 30 μl of 25 mM NH_4_HCO_3_ were added to cover the gel pieces. The trypsin digestion took place at 37 °C for 4 h. The supernatant from the digestion was transferred to a new tube with 5 μl of extraction buffer [50% (*v*/v) ACN/3%(*v*/v) acetic acid]. Gel pieces were extracted twice by adding 35 μl of extraction solution and vortexing for 20 min. Extracts were combined and dried in a speed vacuum. Extracted peptides were resuspended in 7 μl of 0.1% formic acid. Samples were analyzed using liquid chromatography – mass spectrometry (LC–MS) and LC–MS/MS analysis. An aliquot of the digestion mixture (1 μL) was used for LC separation using the Ultimate 3000 RSLCnano UHPLC system with an autosampler (Dionex Corporation, Sunnyvale, California). The eluent was ionized by a nanoelectrospray ionization source of an LTQ Orbitrap XL mass spectrometer (Thermo Scientific, Waltham, Massachusetts). LC–MS data were acquired in an information-dependent acquisition mode, cycling between a MS scan (m/z 310–2000) acquired in the Orbitrap, followed by low-energy collision-induced dissociation (CID) analysis in the linear ion trap. The centroid peak lists of the CID spectra were generated by PAVA [66] and searched against a database that consisted of the National Center for Biotechnology Information (NCBI) protein database, to which a randomized version had been concatenated, using Batch-Tag, a program in the University of California-San Francisco Protein Prospector version 5.10.15. A precursor mass tolerance of 15 ppm and a fragment mass tolerance of 0.5 Da were used for the protein database search. Protein hits were reported with the following parameters: a Protein Prospector protein score of ≥22, peptide score ≥ 15, and E value for protein ≤0.01 [67]. This set of protein identification parameters threshold did not return any substantial false-positive protein hits from the randomized half of the concatenated database. Test samples were compared with corresponding control samples using the Search Compare program.

### Quantitative reverse transcription PCR (qRT-PCR)

The same RNA samples used for RNA sequencing were also used for qRT-PCR. The RNA (400 ng) was transcribed into cDNA using the GoScript™ Reverse Transcriptase (Promega, Madison, Wisconsin) according to the manufacturer’s instructions. The cDNA was quantified by a Nanodrop 2000c spectrophotometer, diluted to 10 ng/μL working stocks in RNase-free H_2_O, and stored at −20 °C until used. Amplification and detection of gene expression were performed using Agilent MP3000 qPCR system (Agilent Technologies, Santa Clara, California). The primers used for these experiments are listed in Additional file 1. Each primer sequence was blasted against the *Frankia* sp. strain CcI6 genome to ensure specificity to the target gene. Standard curves were generated using *Frankia* sp. strain CcI6 genomic DNA and each primer set to test primer efficiency before use. The qRT-PCR experiments were designed in such a way that gDNA contamination could be kept under check. The *rpsO* (CCI6_RS04555) gene was used as the normalizer for all qRT-PCR experiments. The qRT-PCRs were done using 50 ng template cDNA, primer mix (0.3 μM), and SYBR Green PCR Master Mix (Applied Biosystems, Carlsbad, California) in a 25 μL total reaction volume. The following thermal cycler parameters were used: (i) 95 °C for 15 min; (ii) 40 cycles of 95 °C for 15 s and 60 °C for 30 s; and (iii) 1 thermal disassociation cycle of 95 °C for 60 s, 55 °C for 30 s, and incremental increases in temperature to 95 °C for 30 s. Reactions were performed in triplicate. The ∆∆Ct method [68] was used to calculate relative expression (fold changes).

## Results

### Strains CcI6 and Allo2 are highly salt tolerant

**Fig. 1:**
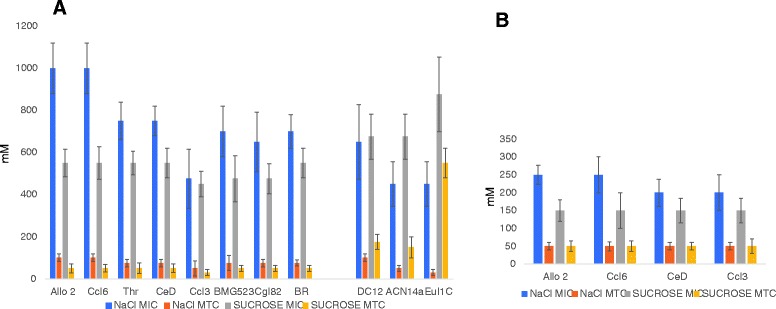
Salt sensitivity assay. **a** The minimum inhibitory concentration (MIC) and maximum tolerance concentration (MTC) values for the different *Frankia* strains exposed to salt (NaCl) and osmotic stress (imposed by sucrose treatment) under nitrogen-sufficient (NH_4_Cl) conditions. For *Casuarina* isolates (left of the bar graph), the MIC and MTC values are expressed as the average values calculated from the dry weight and the BCA protein assays. For the non-*Casuarina* isolates (DC12, ACN14a, and EuI1c), only the dry weight measurements were used to determine the MIC and the MTC values as natural pigments produced by these strains interfered with the BCA protein assay. **b** The MIC and MTC values for salt-tolerant and salt-sensitive *Casuarina* isolates under nitrogen-deficient (N_2_) conditions

The salt tolerance levels for *Frankia* strains isolated from *Casuarina* hosts were measured and compared to the levels found for *Frankia* strains isolated from non-*Casuarina* hosts. Strains CcI6 and Allo2 were highly salt-tolerant and exhibited a NaCl MIC value up to 1000 mM (Fig. 1a). Strain CcI3 was the least salt-tolerant having a MIC value around 475 mM. The other *Casuarina* isolates had intermediate levels of tolerance with MIC values ranging from 650 mM to 750 mM. In general, *Casuarina* isolates had a higher level of salt tolerance compared to other *Frankia* isolates, but this higher level of tolerance was not extended to osmotic stress (Fig. 1a). Here, the other *Frankia* isolates exhibited higher levels of tolerance to osmotic stress compared to the *Casuarina* isolates.

### Salt tolerance is highly dependent on the external supply of nitrogen

Under nitrogen deficient conditions, the level of salt tolerance by *Frankia* strains (including the most salt-tolerant isolates) dramatically decreased (Fig. 1b). For strains CcI6 and Allo2, the NaCl MIC value decreased from 1000 mM under nitrogen sufficient conditions to 250 mM under nitrogen deficient conditions. The observed differences in salt tolerance levels between the salt-tolerant and salt-sensitive strains also substantially decreased under nitrogen deficient conditions (Fig 1b). Vesicle formation and nitrogenase activities were also affected by salt stress. Nitrogenase activity was more severely affected (Additional file 2). When the strains were grown in a medium containing greater than 200 mM NaCl, nitrogenase activity was drastically reduced (Additional file 2).

### Genomic characteristics of *Frankia* strains isolated from *Casuarina* trees

Table 1 presents the genomic features of *Frankia* strains isolated from *Casuarina* trees. The size and G + C content of the genomes used in this study ranged from approximately 5 to 5.6 MB and 69.3 to 70.1% G + C, respectively. Strain CcI3 had the highest number of CDSs (4327), while strain CeD had the lowest number of CDSs (3807).

**Table 1:** Genomic features of *Frankia* strains isolated from *Casuarina* trees

	*Frankia* sp. strains isolated from *Casuarina* trees						
	CcI6	Allo2	CeD	Thr	Br	CcI3	BMG5.23
Chromosome size(Mb)	5.58	5.35	5.00	5.31	5.23	5.43	5.27
GC %	69.3	70.0	70.0	70.0	70.0	70.1	69.9
N_50_ (bp)	103, 000	96,900	73,600	71,600	60,200	543,3628	64,900
CDS	4280	4224	3857	4209	4220	4327	4114
rRNA	9	8	6	5	4	6	9
tRNAs	45	45	45	45	45	45	48
#Scaffolds	136	110	120	169	180	1	166
#Contigs	155	133	154	184	180	1	191
Reference	[17]	[21]	[18]	[16]	[19]	[20]	[15]

**Fig. 2:**
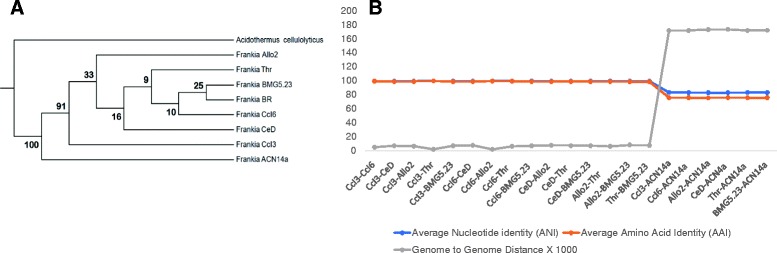
Genomic taxonomy of *Frankia* strains isolated from *Casuarina* spps. **a** Concatenated phylogenetic affiliation of 394 maximum-parsimony trees for amino acid sequences of orthologs among all of the genomes including *Casuarina* isolates, *F. alni* strain ACN14a isolated from *Alnus* and *Acidothermus celloluyticus,* which was used as an outgroup. The numbers on the branches represent the percent confidence of speciation of a given branch. **b** AAI, ANI, and genome to genome distance (multiplied by 1000) values for the different *Casuarina* isolates and *F. alni* strain ACN14a isolated from *Alnus* which is included for comparison. Genome to genome distance was determined by GGDC as a function of sum of all identities found in HSPs divided by overall HSP length. GGDC2 BLAST+ was used as the alignment method for finding intergenomic matches

We obtained a phylogeny of the *Frankia* strains from a set of 394 conserved non-duplicated orthologs (Fig. 2a). As expected, the eight *Casuarina* strains grouped together and were distinct from the closely related Cluster-1a strain ACN14a, which was isolated from *Alnus* trees. Strains CcI6 and Allo2 showed close similarity and grouped together, while strains CeD and BMG5.23 showed the least similarity with other strains.

### Average nucleotide identity, average amino acid identity, and genome to genome distance

The average nucleotide identity between any pair of *Frankia* strains isolated from *Casuarina* trees was greater than 99%. In contrast, the average nucleotide identity between any *Casuarina* isolate and the closely-related strain ACN14a was less than 85% (Fig. 2b). Similar results were obtained for the average amino acid identity and genome-to-genome distance values. Based on DNA-DNA hybridization (DDH) prediction by GGD 2.1, at *p = 0.05* level, any two *Casuarina* isolates have at least 70% DDH value, the cutoff point for species delineation. On the other hand, in a pairwise comparison with strain ACN14a, none of the *Casuarina* isolates had DDH value greater than or equal to 70% at *p* = 0.05 level.

### Pan-genome analysis reveals a high abundance of singletons among all of the strains

Pan-genome analysis was performed by orthologous clustering using OrthoVenn. OrthoVenn utilizes OrthoMCL to perform an all-against-all BLASTP alignment and identify putative orthology and paralog relationships with the Inparanoid algorithm.

**Fig. 3:**
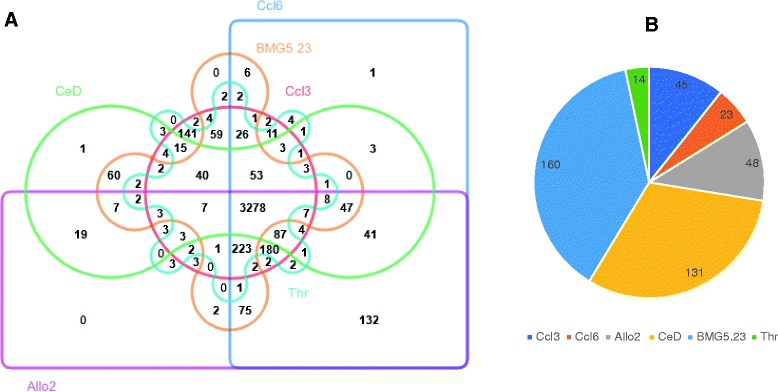
Pangenome overview of six *Frankia* strains isolated from *Casuarina* trees. **a** Six-way Venn diagram showing shared and specific gene clusters among the *Casuarina* isolates as determined by OrthoVenn. An E-value cutoff of 1 e^−5^ was used for protein similarity search and inflation value of 1.5 was used for the generation of orthologous clusters using the Markov Cluster Algorithm. **b** Number of singletons identified in each *Casuarina* isolate

The OrthoVenn analysis of the six *Casuarina* isolates selected after the salt sensitivity assay revealed 3278 pan-orthologous gene clusters, of which 3246 were single copy pan orthologous gene clusters (Fig. 3a). Pairwise comparison of the genomes showed that strains CcI6 and Allo2 shared the highest number of unique clusters (132), not found in the other strains (Additional file 3). No genome had more than 2 clusters unique to itself, but all had many singletons (Fig. 3b) suggesting that there was insufficient time for gene duplication events to occur after the appearance of singletons. Among the six *Casuarina* isolates, strain BMG5.23 had the highest number of singletons (160). About 30% of the singletons in any one strain were hypothetical proteins. Singletons generally occur dispersed within the genome, suggesting they were acquired independently. However, in strain BMG5.23, singletons seem to be clustered in the same region. The singletons in strain BMG5.23 had varying GC contents and the fact that at least some of them co-occur in the same region suggests that there might be hot spots for insertion.

### The two salt-tolerant strains contain many hypothetical proteins absent in the other strains

**Fig. 4:**
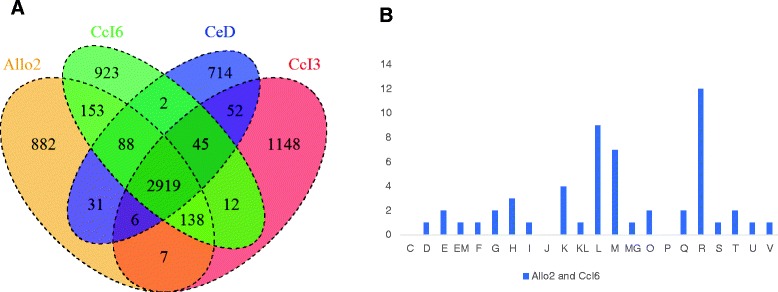
Pangenome analysis of single-copy genes (**a**) Shared and specific single-copy orthologous CDSs among the highly salt-tolerant (Allo2 and CcI6), the moderately salt-tolerant (CeD), and the salt sensitive (CcI3) strains. **b** Distribution of the 153 single-copy genes specific to the two highly salt-tolerant strains into functional COG categories: C, energy production and conversion; D, cell division and chromosome partitioning; E, amino acid transport and metabolism; F, nucleotide transport and metabolism; G, carbohydrate transport and metabolism; H, coenzyme metabolism; I, lipid transport and metabolism; J, translation, ribosomal structure and biogenesis; K, transcription; L, DNA replication, recombination and repair; M, cell wall/membrane biogenesis; O, posttranslational modification, protein turnover, chaperones; P, inorganic ion transport and metabolism; Q, secondary metabolite biosynthesis, transport and catabolism; R, general function prediction only; S, function unknown; T, signal transduction mechanisms; U, intracellular trafficking and secretion; and V, defense mechanisms. Proteins that could be classified to more than one category are represented by two letters

While genomic comparison of the salt-tolerant strains (CcI6 and Allo2) against the entire *Casuarina*-associated strains gave insight on the strain difference, this analysis included multiple gene copies. Comparison of the single copy orthologous gene clusters between the salt-sensitive isolate (CcI3), the moderately salt-tolerant strain (CeD), and the two highly salt-tolerant strains (CcI6 and Allo2) was also performed using the Lerat program. All four strains shared 2919 single copy core genes (Fig. 4a). The two highly salt-tolerant strains contained 153 single copy orthologous genes that were not shared with the moderately-tolerant and salt-sensitive strains (Additional file 3). Both highly salt-tolerant strains and the moderately-tolerant strain shared 88 single copy genes that were not present in the salt-sensitive strain. Figure 4b shows the distribution of genes found in the two highly salt-tolerant strains into Cluster of Orthologous Groups of protein (COG) functional categories. Among the 153 unique genes found in the two salt-tolerant strains, 114 of the genes were annotated as hypothetical proteins. However, re-annotation using the RPSBLAST program on the COG database (prokaryotic proteins) revealed only 99 hypothetical proteins. The three COG categories that were highly represented among the unique genes found only in the two highly salt-tolerant strains were: (COG R) general function prediction only; (COG L) DNA replication, recombination, and repair; and (COG M) cell wall/membrane envelope biogenesis. Tolerant-strain-specific genes assigned to the cell wall/membrane biogenesis (COG M) category include genes encoding glycosyl transferases, proteins involved in cellulose synthesis, D-alanine: D-alanine ligase (Ddl), and a predicted nucleoside-diphosphate-sugar epimerase. Glycosyl transferases allow for a more flexible response to environmental stress. In tobacco, ectopic expression of a glycosyl transferase (UGT85A5) leads to enhanced salt tolerance [69]. Ddl is involved in the D-alanine branch of peptidoglycan biosynthesis. Mutation in a D-alanine-D-alanine ligase of *Azospirillum brasilense* Cd results in an overproduction of exopolysaccharides and decreased tolerance to saline stress [70].

Another COG functional category that was represented among the genes found only in the tolerant strains was coenzyme metabolism. Two genes encoding hypothetical proteins (CCI6_RS06345, CCI6_RS15790) with ubiquinone synthesis-related methyl transferase domains and a hypothetical protein with a geranylgeranyl pyrophosphate synthase domain (CCI6_RS02885) were among the tolerant strain-specific genes assigned to the coenzyme metabolism functional category. Geranylgeranyl pyrophosphate (GGPS) synthase catalyzes formation of geranylgeranyl pyrophosphate (GGP), which is a key step in the biosynthetic pathway of carotenoïds and many other terpenes [71].

### Salt-tolerant and -sensitive strains contain the same set of classical salt-tolerance genes

Many of the known salt tolerance mechanisms are also osmotic stress response mechanisms or vice versa. The *Casuarina* associated *Frankia* genomes were data mined for the presence of these known osmotic/salt tolerance mechanisms.

All of these genomes had a *kdpFABCDE* operon encoding the membrane-associated P-type ATPase, Kdp-ATPase (*kdpFABC*), involved in K^+^ uptake and a two-component regulatory system (*kdpDE*), which regulates the expression of *kdpFABC* [72]. The Kdp system plays a role in ion homeostasis and adaptation to osmotic stress. Both tolerant and sensitive strains also contained the Trk system, which is the predominant uptake system in medium containing more than 1 mM K^+^. All of the *Casuarina* associated *Frankia* genomes lacked mechanisms for *de novo* synthesis or uptake of glycine betaine. However, all of the *Casuarina* isolates possessed the capacity for the biosynthesis of the important osmo-protectant proline. Three pathways for the synthesis of trehalose, an effective osmolyte, were also present in all the *Casuarina* associated *Frankia* genomes. The first pathway (the TreY-TreZ pathway) synthesizes trehalose from glycogen-like alpha (1-- > 4)-linked glucose polymers. The second pathway (the TreS pathway) synthesizes trehalose from maltose, while the third pathway, the OtsA-OtsB pathway, utilizes glucose-6-phosphate and UDP-glucose to synthesize trehalose through a two-step enzymatic process involving trehalose-6-phosphate synthase (OtsA) and trehalose-6-phosphate phosphatase (OtsB). All *Casuarina* isolates contained the *asnO–ngg* cluster putatively involved in the synthesis of N-acetylglutaminylglutamine amide (NAGGN), a dipeptide identified as an osmolyte in a few bacteria. The first step of the reaction involves the N-acetylation of a glutamine residue and the subsequent dipeptide bond formation between this residue and a second L-glutamine residue in a reaction catalyzed by Ngg. In the second step of the reaction catalyzed by AsnO, an amide group is transferred from a free L-glutamine molecule to the second L-glutamine residue of NAGG to produce NAGGN. Just like most other genomes containing the *asnO–ngg* cluster, the genomes of all *Casuarina* isolates encode a dipeptidase immediately downstream of the *ngg* gene. A possible role for such a peptidase could be balancing of the NAGGN pool during adaptation to osmotic fluctuations. The identities between AsnO from *Casuairna* isolates and the one found within other bacterial species was high (greater that 60%) whereas the putative Ngg protein from *Casuarina* isolates had low identity (< 15%) with Ngg proteins identified in other species. In contrast to the *asnO-ngg* organization found within other genomes, the *asnO* and the *ngg* genes in the *Casuarina* isolates were not contiguous, but had the dipeptidase gene between them.

### Transcriptome analysis of the highly salt-tolerant strain (CcI6) reveals hundreds of genes that are responsive to salt stress

After identifying unique genes in the two highly salt tolerant strains through comparative genomics, we attempted to identify the role of these unique genes in the response to salt and osmatic stress. This was done by transcriptome profiling of strain CcI6, one of the two highly salt-tolerant strains, under salt or osmotic stress and comparing to the profile under control conditions (no stress). Strain CcI6 was exposed to either no stress, salt stress or osmotic stress for 7 days and the transcriptome profile was analyzed using RNA-seq. Two biologically independent experiments were performed for each condition. After sequencing of the libraries, an average of 17 million, 9.5 million, and 19.8 million reads were obtained under the control, salt stress, and osmotic stress conditions, respectively. CLC Genomics Workbench 9.0.1 was used to map the reads to the annotated *Frankia* sp. CcI6 genome. An average of 2.14 million, 0.85 million, and 2.5 million read pairs could be unambiguously mapped to the CDSs for the reference condition, for the salt stress condition, and for the osmotic stress condition, respectively. Based on the mapped reads, coverage for the samples were 42×, 17×, and 100× for the control, salt stress and osmotic stress conditions, respectively.

**Fig. 5:**
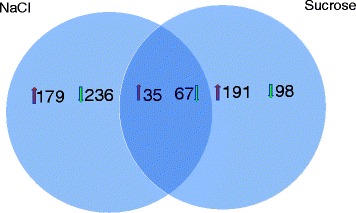
Global gene expression responses following salt and osmotic stress. Venn diagram showing the extent of overlap between genes differentially expressed under salt and osmotic stress in the salt-tolerant strain *Frankia* sp. CcI6. The arrows indicate the number of up-regulated and down-regulated genes under salt and osmotic stress. The intersection indicates the number of differentially expressed genes under both conditions

Transcriptome analysis revealed that a total of 214 and 226 genes were up-regulated and 303 and 167 genes were down-regulated under salt and osmotic stress, respectively. Thirty-five up-regulated and 65 down-regulated genes were found in common with both conditions (Fig 5). The complete list of differentially expressed genes is provided in Additional file 4. Among the genes up-regulated under both salt and osmotic stress were hypothetical proteins, proteins involved in cell wall/membrane biogenesis functions and the following transport proteins: an ABC-type Fe3+ hydroxamate transport system, periplasmic component (CCI6_RS09145) and ABC-type Fe3+ siderophore transport system, permease component (CCI6_RS09155). This result suggests that increased iron uptake is part of the general response to salt and osmotic stresses. Increased iron uptake under salt stress has been reported for *Bacillus subtilis* [73].

**Fig. 6:**
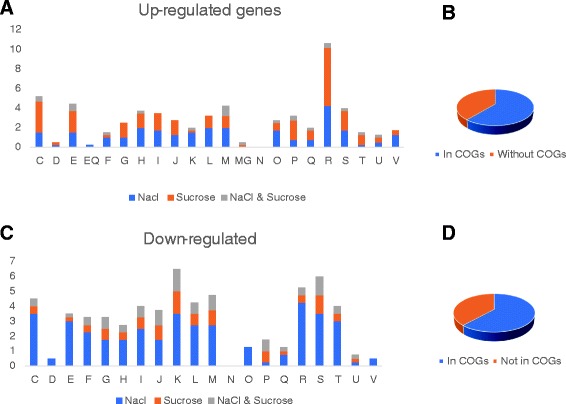
The percentage of the differentially expressed genes that fell within the various Clusters of Orthologous Gene (COG) categories. The columns are labelled as follows: C, energy production and conversion; D, cell division and chromosome partitioning; E, amino acid transport and metabolism; F, nucleotide transport and metabolism; G, carbohydrate transport and metabolism; H, coenzyme metabolism; I, lipid transport and metabolism; J, translation, ribosomal structure and biogenesis; K, transcription; L, DNA replication, recombination and repair; M, cell wall/membrane biogenesis; N, cell motility; O, posttranslational modification, protein turnover, chaperones; P, inorganic ion transport and metabolism; Q, secondary metabolite biosynthesis, transport and catabolism; R, general function prediction only; S, function unknown; T, signal transduction mechanisms; U, intracellular trafficking and secretion; and V, defense mechanisms. For each condition, the number of up-regulated (or down-regulated) genes in each COG category was expressed as a percentage total number of upregulate or down-regulated genes, respectively. **a** Up-regulated genes. **b** Fraction of up-regulated genes that are assigned to COG categories. **c** Down-regulated genes. **d** Fraction of down-regulated genes that are assigned to COG categories

A total of 179 genes were only up-regulated under salt stress, while 191 genes were only up-regulated under osmotic stress (Fig 5). The functional category analysis of the up-regulated and down-regulated genes showed that more than 60% and 61% of the genes, respectively, were assigned into a COG functional category (Fig. 6b, d). Unassigned genes were mainly comprised of hypothetical proteins. COG functional categories highly represented among up-regulated genes included: (1) general function prediction only (COG R), energy production and conversion (COG C), amino acid transport and metabolism (COG E) [Fig 6a]. Among the up-regulated genes, 3.7% and 5.5% of the genes were general function prediction only (COG R) genes specific to salt and osmotic stress, respectively. General function prediction only (COG R) genes up-regulated under both salt and osmotic stress constituted 0.5% of the total up-regulated genes. A total of 2% and 1.2% of the up-regulated genes were cell wall/membrane biogenesis (COG M) genes specific to salt and osmotic stress, respectively (Fig 6a). One percent of the up-regulated genes comprised of cell wall/membrane biogenesis (COG M) genes up-regulated under both salt and osmotic stress conditions. Energy production and conversion (COG C) genes comprised 4.7% of the total up-regulated genes. Among the up-regulated genes assigned to COG C, roughly 23% were specific to salt stress while 65% were specific to osmotic stress and 12% were common to both salt and osmotic stress conditions. Functional categories that are highly represented among down-regulated genes include transcription (COG K), general function prediction only (COG R), and function unknown (COG S). Salt stress-specific and osmotic stress-specific transcription (COG K) genes constitute 2% and 0%, respectively of the down-regulated genes. Transcription (COG K) genes that are down-regulated under both salt and osmotic stress conditions make up 1.5% of the total down-regulated genes.

We validated the RNA-seq data by performing quantitative reverse transcription PCR (qRT-PCR) on 11 genes selected from the RNA-Seq analysis (Table 2). A high degree of correlation (*R* = 0.95) was observed between the normalized values of the fold change from the qPCR data and the normalized fold change values from the RNA-Seq data.

**Table 2:** qRT-PCR validation of RNA-seq data

Locus tag	qRT-PCR fold change (salt stress vs control)	RNAseq fold change (salt stress vs control)	RNAseq vs qRT-PCR *p-value* (salt stress)	qRT-PCR fold change (osmotic stress)	RNAseq fold change (osmotic stress)	RNAseq vs qRT-PCR *p-value* (osmotic stress)
CCI6_RS21730^a^	2.100^e^	2.360	0.840	1.100	−6.000	0.012^d^
CCI6_RS17915^a^	3.120^e^	3.240	0.960	1.820^e^	1.980	0.870
CCI6_RS19875^a^	2.500^e^	4.910	0.03^d^	3.011^e^	11.210	0.023^d^
CCI6_RS17580^a^	3.140^e^	3.480	0.870	2.120^e^	1.520	0.560
CCI6_RS16000^a^	1.890^e^	2.183	0.930	−0.500	−1.200	0.040^d^
CCI6_RS18570^c^	1.320	1.230	0.240	1.511	1.380	0.720
CCI6_RS06495^c^	1.400	1.500	0.450	−1.310	−2.020	0.220
CCI6_RS02325^b^	3.500^e^	3.840	0.830	2.800^e^	3.860	0.620
CCI6_RS12340^c^	−1.300	−4.000	0.023^d^	1.300	−9.000	0.026^d^
CCI6_RS08505^a^	3.230^e^	3.590	0. 753	1.150	1.051	0.972
CCI6_RS19950^b^	10.021	12.061	0.778	18.231	21.585	0.812

^a^Up-regulated genes under salt stress according to RNA-Seq analysis

^b^Up-regulated genes under both conditions according to RNA-Seq analysis

^c^Non-differentially expressed genes in the study according to RNA-Seq analysis

^d^Indicates that there is a statistically significant difference between fold change values determined by qPCR and RNA-seq

^e^Indicates a statistically significant qRT-PCR fold change value

The fold change values (salt vs control or sucrose vs control) determined from the RNAseq experiment were compared with the fold change values obtained from qRT-PCR experiments. For the qRT-PCR experiments, the ∆∆C_t_ method was used to determine the fold change. The *rpsA* gene was used as the normalizer for all of the qRT-PCR experiments. The fold change values for the RNA-seq experiments are based on two biological replicates whereas the fold change values for the qRT-PCR experiments are based on three biological replicates

### Many hypothetical proteins that are unique to the tolerant strain were up-regulated under salt stress

Under osmotic stress, an acetyl transferase with general function prediction only and 7 hypothetical proteins that are unique to the two salt-tolerant strains were up-regulated. Under salt stress, a zinc peptidase, ADP-heptose:LPS heptosyltransferase, and 5 hypothetical proteins unique to the tolerant strains were up-regulated (Table 3). One hypothetical protein (CCI6_RS13590) was up-regulated under both salt and osmotic stress conditions. In a study of differentially expressed genes in salt-tolerant and salt sensitive varieties of rice, zinc peptidase was among the 50 highest responsive genes in the salt-tolerant variety [74].

**Table 3:** Genes unique to the tolerant strain with increased expression under salt stress

Salt Stress			Osmotic Stress		
Locus Tag	Protein product	COG category	Locus Tag	Protein product	COG category
CCI6_RS13590	Hypothetical Protein	–	CCI6_RS13590	Hypothetical Protein	–
CCI6_RS22605	Predicted Zn peptidase	E	CCI6_RS17905	Acetyltransferase (isoleucine patch superfamily)	R
CCI6_RS17915	ADP-heptose:LPS heptosyltransferase	M	CCI6_RS13555	Hypothetical Protein	–
CCI6_RS19875	Hypothetical Protein	–	CCI6_RS15755	Hypothetical Protein	–
CCI6_RS20860	Hypothetical Protein	–	CCI6_RS21770	Hypothetical Protein	–
CCI6_RS17580	Hypothetical Protein	–	CCI6_RS13535	Hypothetical Protein	–
CCI6_RS21730	Hypothetical Protein	–	CCI6_RS02120	Hypothetical Protein	–

### Versatile responses of transcription factors

Not surprisingly, COG K (transcription) genes were highly represented in the transcriptome of strain CcI6 under salt stress (Additional file 4). Under salt stress, 6 genes (*CCI6_RS12535, CCI6_RS20460, CCI6_RS18600, CCI6_RS15305, CCI6_RS01570, CCI6_RS12900, CCI6_RS02550*) encoding transcriptional factors from the GntR, TetR, and LysR, and the Crp/Fnr families and 6 genes (*CCI6_RS00475, CCI6_RS02550, CCI6_RS10900, CCI6_RS11405, CCI6_RS17055, CCI6_RS21525*) encoding transcriptional regulators from Crp/Fnr and LuxR families were up-regulated under salt and osmotic stress, respectively. One transcriptional regulator belonging to the Crp/Fnr family (CCI6_RS02550) was up-regulated under both salt and osmotic stress conditions. Transcriptional factors belonging to GntR, TetR, LysR, and the Crp/Fnr families have been implicated previously in several stress responses including heat and osmotic shock [75]. In addition, only one (CCI6_RS19210) of the 12 sigma factors present in strain CcI6 was up-regulated under salt stress, while another one, an extracytoplasmic stress sigma factor (CCI6_RS15595), was down-regulated under salt stress conditions.

### Salt stress up-regulated several genes involved in peptidoglycan modification

Two genes encoding polysaccharide deacetylases (*CCI6_RS03540, CCI6_RS11155*) were up-regulated under salt stress, but were unchanged under osmotic stress (Additional file 4). In *Bacillus anthracis*, a polysaccharide deacetylase plays a role in the adaptation of the bacteria to a high salt environment [76]. Under salt stress only, 4 glycosyl transferases (*CCI6_RS10910, CCI6_RS07965, CCI6_RS21895, CCI6_RS01640*) associated with cell wall/membrane biogenesis showed more than 4-fold increase. Similarly, under osmotic stress, 4 glycosyl transferases associated with cell wall/membrane biogenesis (*CCI6_RS02325, CCI6_RS10910, CCI6_RS11195, CCI6_RS11220*) were up-regulated. One of the above glycosyl transferase genes (*CCI6_RS10910*) showed a statistically significant up-regulation under both salt and osmotic stress.

Three nucleoside diphosphate sugar epimerase genes (*CCI6_RS19225, CCI6_RS08005, CCI6_RS04525*) were up-regulated under salt stress, while two nucleoside diphosphate sugar epimerases (*CCI6_RS00960, CCI6_RS08005*) were up-regulated under osmotic stress. Together, these results suggest that cell wall modifications are involved in the response to salt stress and osmotic stress.

### Modulation of membrane composition

Two genes encoding acyl-acyl carrier protein (ACP) desaturases (*CCI6_RS10965, CCI6_RS10965*), were up-regulated only under salt stress, but not under osmotic stress (Additional file 4). In addition to desaturases, two other genes [acyl-CoA dehydrogenase (*CCI6_RS05135*) and ABC-type branched-chain amino acid transport systems, periplasmic component (*CCI6_RS10620*)] that help determine membrane fluidity were up-regulated under salt stress. Furthermore, salt stress caused the up-regulation of *ubiE* (*CCI6_RS17660*) gene encoding a multispecies ubiquinone biosynthesis protein. Ubiquinone accumulation has been shown to increase salt tolerance in *E. coli* through mechanical stabilization of the membrane [77]. Taken together, these results suggest that *Frankia* membrane fluidity was altered by salt stress.

### Different osmolytes might be used by the cell depending on the external solute causing the stress

The trehalose synthase gene (*CCI6_RS13*215) was up-regulated under salt stress, while the glutamate synthase (*CCI6_RS10225*) and threonine synthase (*CCI6_RS08750*) genes were up-regulated under osmotic stress. The regulation of intracellular osmolality by the transport or biosynthesis of compatible solutes is believed to be the principal osmoprotection response in bacteria.

### Proteomics analysis reveals additional functions that might be involved in salt stress tolerance

**Fig. 7:**
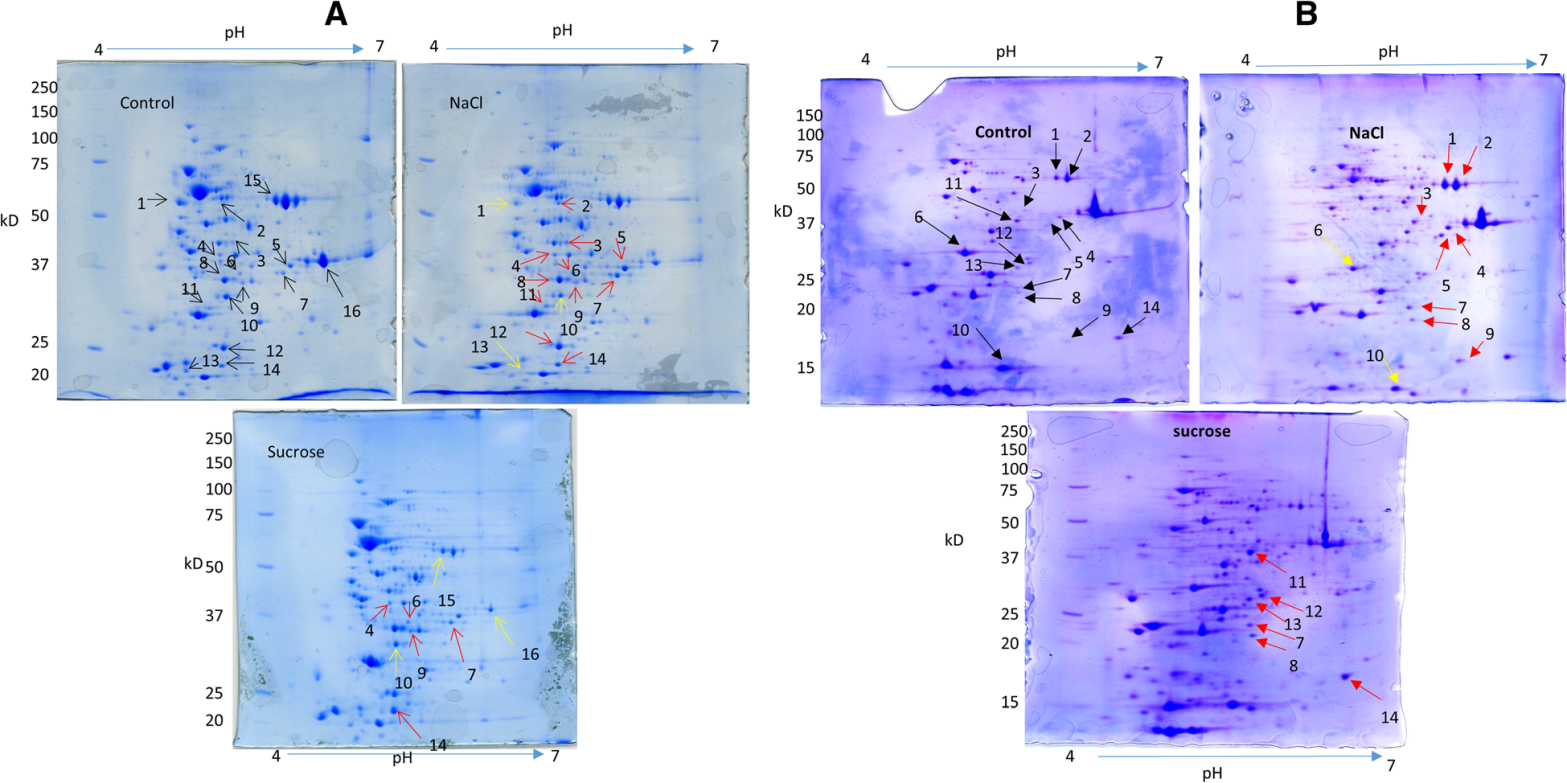
Two-dimensional polyacrylamide gel electrophoresis (PAGE) analysis of salt stress response*.* **a** 2D–gel profile of *Frankia* sp. strain CcI6 under control (no stress), 200 mM NaCl, and 200 mM sucrose stress conditions. **b** 2D–gel profile of *Frankia casurainae* strain CcI3 under control (no stress), 200 mM NaCl, and 200 mM sucrose stress conditions. Red arrows indicate that proteins are upregulated relative to the control, while yellow arrows indicate down regulated proteins relative to the control. The corresponding number spots were in-gel digested with trypsin and analyzed by liquid chromatography-mass spectrometry (LC-MS) and LC-MS/MS for protein identification

The proteome profile of the salt-tolerant (CcI6) and salt sensitive (CcI3) strains exposed to salt or osmotic stress was examined by the use of 2D SDS-PAGE (Fig. 7). For both strains, prominent changes in protein abundance were readily noticeable under the three different conditions (no stress, salt stress and osmotic stress) and occurred in multiple replicates.

Differential protein spots were screened and those that showed the most prominent differences between the stress conditions and control gels were targeted for further analysis. For the salt-tolerant strain, 16 differentially expressed spots for all three conditions were analyzed and 19 proteins were identified. For the salt-sensitive strain, 14 differentially expressed spots for all three conditions were analyzed and 8 proteins were identified. For strain CcI6, eleven and three spots were upregulated and downregulated, respectively, under salt stress, while five and three spots were upregulated and downregulated, respectively, under osmotic stress. Out of the 19 salt-responsive strain CcI6 proteins, 18 were assigned into COG functional categories, including energy production and conversion (COG C, 4 proteins), transcription (COG K, 3 proteins), amino acid transport and metabolism (COG E, 2 proteins), post translational modification, protein turn over, chaperon functions (COG O, 2 proteins), carbohydrate transport and metabolism (COG G, 2 proteins) [Table 4].

**Table 4:** Proteins differentially expressed under stress conditions in strains CcI6 and CcI3

	SPOT #	Acc. No	Locus Tag	Protein Name	MW (Da)	PI	NaCl	Sucrose
[C] Energy production and Conversion								
CcI6	10	563,313,506	CCI6_RS13315	electron transfer flavoprotein alpha subunit apoprotein	32,842.70	5.01	↓	↓
	15	563,312,797	CCI6_RS16290	NAD-dependent aldehyde dehydrogenase	54,456.60	5.40	N/C	↓
	1	563,313,455	CCI6_RS13080	ATP synthase F1 subcomplex beta subunit	56,735.20	4.56	↓	N/C
	8	563,314,955	CCI6_RS05745	malate dehydrogenase (NAD)	34,399.10	4.96	↑	N/C
[E] Amino acid transport and metabolism								
CcI6	2	563,312,117	CCI6_RS19490	L-glutamine synthetase	53,786.20	4.97	↑	N/C
	10	563,315,075	CCI6_RS05150	cysteine synthase (CysK)	32,443.60	4.95	↑	↓
[G] Carbohydrate transport and metabolism								
CcI6	5	563,312,326	CCI6_RS18410	glyceraldehyde-3-phosphate dehydrogenase (NAD+)	35,515.80	5.76	↑	N/C
	7	563,314,408	CCI6_RS08915	fructose-bisphosphate aldolase	36,894.20	5.35	↑	↑
CcI3	4	WP_011436076.1	FRANCCI3_RS08225	glyceraldehyde-3-phosphate dehydrogenase (NAD+)	35,515.80	5.76	↑	N/C
	5	WP_011438718.1	FRANCCI3_RS22085	fructose-bisphosphate aldolase	36,894.20	5.35	↑	N/C
	11	WP_011437192.1	FRANCCI3_RS14110	aldolase	38,526.7	5.3	N/C	↑
[H] Coenzyme metabolism								
CcI6	3	563,314,085	CCI6_RS10325	methionine adenosyltransferase	42,893.60	5.05	↑	N/C
	9	563,313,788	CCI6_RS11580	pyridoxal phosphate synthase yaaD subunit	32,577.80	5.34	↑	↑
CcI3	9	WP_011437570.1	FRANCCI3_RS16030	6,7-dimethyl-8-ribityllumazine synthase	16,137.8	5.5	↑	N/C
[I] Lipid transport and metabolism								
CcI3	3	WP_011438063		acetyl-CoA acetyltransferase	39,714.5	5.3	↑	N/C
[J] Translation								
CcI3	8	WP_011437955.1	FRANCCI3_RS18065	ribosome-recycling factor	20,830.7	5.4	↑	↑
[K] Transcription								
CcI6	3	563,314,632	CCI6_RS07960	DNA-directed RNA polymerase, sigma subunit (sigma70/sigma32)	44,399.30	5.10	↑	N/C
	12	563,312,999	CCI6_RS15595	RNA polymerase, sigma 24 factor	29,218.90	5.49	↓	N/C
	13	563,314,238	CCI6_RS09105	Transcriptional regulator Crp/Fnr	51,393.10	4.97	↓	N/C
CcI3	14	WP_011437589.1	FRANCCI3_RS16125	XRE family transcriptional regulator	17,993.4	5.7	↑	N/C
[M] Cell wall/membrane/envelop biogenesis								
CcI6	6	563,313,716	CCI6_RS12035	UDP-glucose pyrophosphorylase	34,856.10	5.04	↑	↑
	11	563,315,562	CCI6_RS03270	Nucleoside-diphosphate-sugar pyrophosphorylase family protein	31,492.90	4.92	↑	N/C
[O] Post-translational modification, protein turnover, chaperone functions								
CcI6	2	563,314,374	CCI6_RS08745	chaperonin GroEL	56,735.20	4.72	↑	N/C
	14	563,311,297	CCI6_RS22940	ATP-dependent Clp protease proteolytic subunit ClpP	23,039.30	4.79	↑	↑
[R] General Functional Prediction only								
CcI6	16	563,312,796	CCI6_RS16285	Zn-dependent alcohol dehydrogenase	35,062.50	5.65	N/C	↓
CcI3	7	WP_011434678.1	FRANCCI3_RS01050	FMN reductase	21,222.1	5.3	↑	↑
Not assigned to COG categories								
CcI6	4	563,313,603	CCI6_RS12255	Nitroreductase	37,351.40	4.90	↑	↑
	12	563,313,563	CCI6_RS12810	proteasome endopeptidase complex	28,833.80	5.11	↑	N/C

The identified proteins were classified by COG functional categories. More than one protein per spot has been identified for some spots. Upregulated proteins are shown by the upward pointing arrow (↑) whereas downregulated proteins are shown by the downward pointing arrow (↓). No change (N/C) indicates that a spot was not picked for that particular condition because it showed similar intensity as the control

Among the proteins upregulated in the salt-tolerant strain was a cysteine synthase (CysK) which was identified from a downregulated spot under salt stress (spot no. 10) but had 2.5 times more peptide counts in the NaCl-treated samples. The most abundant protein from the same spot (spot no. 10) was an electron transfer flavoprotein alpha subunit apoprotein (CCI6_RS13315), which, as expected, had a lower peptide count in the NaCl-treated samples. Several pyridoxal phosphate-binding proteins, including cysteine synthase, are differentially expressed under salt stress in wheat chloroplasts and help in the synthesis of cysteine as a protective measure against toxic ions [78]. Transcriptomics analysis of the salt-tolerant strain (CcI6) revealed that CysK and CysM were upregulated under salt stress. CysK and CysM are pyridoxal phosphate-dependent enzymes. Our proteomics result showed that pyridoxal phosphate synthase yaaD subunit (WP_011435808.1) was upregulated under salt stress in strain CcI6. Another protein from the COG E category that was upregulated under salt stress in strain CcI6 was the glutamine synthetase (WP_011437527.1). Amino acids including proline and glutamine, serve as protective osmolytes under salt stress [25]. The transcriptome analysis of CcI6 showed that glutamine synthetase was upregulated 1.4-fold times, but the change was not statistically significant. A GroEL chaperon (WP_011438752.1) and an ATP-dependent Clp protease proteolytic subunit ClpP (WP_011435649.1) were among the COG O proteins upregulated under salt stress in strain CcI6. In *B. subtilis*, the synthesis of ClpP protein increases during heat shock, salt and oxidative stress, glucose and oxygen deprivation [79]. Our transcriptome data also showed that the GroEL chaperon (CCI6_RS08745) and ClpP (CCI6_RS22940) are upregulated in salt-stressed CcI6. Another ClpP protein (ClpP_2) [CCI6_RS22535] was upregulated under osmotic stress in strain CcI6 based on the RNA-seq data, but this protein was not identified in our proteomics data. GroEL is one of the heat shock proteins and shows upregulation under salt stress in *Lactococcus lactis*, suggesting there is overlap between salt and heat stress responses [80]. In strain CcI6, among differentially expressed proteins identified in the COG K functional category, a DNA-directed RNA polymerase sigma subunit (WP_023840564.1) was upregulated under salt stress. The transcriptome analysis did not reveal upregulation of the sigma factor, suggesting posttranscriptional regulation of the transcript. An extracytoplasmic function (ECF) family RNA polymerase, sigma subunit (WP_035729933.1) was downregulated under salt stress in strain CcI6. Our transcriptome analysis also showed downregulation of the ECF family sigma factor. The ECF sigma factors are small divergent group of regulatory proteins that control the transcription of genes associated with response to extracytoplasmic stress conditions and some aspect of the cell surface or transport [81]. Two proteins, glyceraldehyde-3-phosphate dehydrogenase and fructose-bisphosphate aldolase, belonging to the carbohydrate transport and metabolism functional category (COG G), were upregulated under salt stress in strain CcI6. Overexpression of glyceraldehyde-3-phosphate dehydrogenase in rice plants improved salt tolerance [82]. Similarly, the overexpression of fructose-bisphosphate aldolase in *Brassica napus* led to increased salt stress tolerance [83]. Another salt-tolerant strain, Allo2, showed similar changes in the proteome prolife as strain CcI6 under salt and osmotic stress conditions (Additional files 5,6).

For strain CcI3, seven and three proteins were identified from upregulated spots under salt and osmotic stress conditions, respectively. All of the proteins upregulated under osmotic stress were also upregulated under salt stress. CcI3 proteins upregulated under salt stress include glyceraldehyde-3-phosphate dehydrogenase (COG G), fructose-bisphosphate aldolase (GOC G), ribosome-recycling factor (COG J), aldolase (COG G), XRE family transcriptional regulator (COG K), 6,7-dimethyl-8-ribityllumazine synthase (COG H), FMN reductase (COG R), and acetyl-CoA acetyltransferase (COG I). Two of the proteins upregulated under salt stress in CcI3, glyceraldehyde-3-phosphate dehydrogenase and fructose-bisphosphate aldolase, show a similar pattern of upregulation in all three strains (CcI3, CcI6, and Allo2).

## Discussion

### Salt tolerance mechanisms depend on the availability of nitrogen

The salt tolerance levels for strains Allo2 and CcI6 (1000 mM) were comparable to the 500–1500 mM NaCl tolerance reported for moderately halophilic gram-positive bacteria [84]. Strain CcI3 exhibited about half of the salt tolerance level (475 mM) of the two salt-tolerant strains, only slightly less than the level (528 mM) for *Rhizobium meliloti*, a salt-tolerant rhizobium species [85]. Non-*Casuarina* isolates in general, and strain EuI1C in particular, outperformed the *Casuarina* isolates under osmotic stress imposed by sucrose. The results suggest that *Casuarina* isolates have developed a mechanism that specifically copes with the toxic effects of Na^+^ and Cl^−^ ions. This situation also occurs with *Calobacter* [86]*.* The observed difference in salt tolerance levels between salt-tolerant and salt-sensitive *Casuarina* isolates dissipated under nitrogen-deficient (N_2_) conditions, suggests that salt stress response is reliant on ample supply of nitrogen sources. The result is expected as many of the osmotic adjustments that take place -from protective osmolyte synthesis to cell envelope remodeling- rely heavily on nitrogen containing compounds. Under nitrogen-deficient conditions, survival of *Frankia* is dependent on the activities of the nitrogenase enzyme, which reduces atmospheric dinitrogen into ammonia. For all *Casuarina* isolates, both nitrogenase activity and vesicle number per milligram of protein decreased with increasing levels of salinity, causing a compound effect on nitrogen fixation. The fact that nitrogenase activity was affected more severely than overall growth with increasing concentrations of NaCl, and the fact that external nitrogen supply dramatically improves the salt tolerance of strains suggests that the ability to maintain nitrogen fixation under salt-stress is important for salt stress tolerance.

### All of the *Casuarina* isolates are the same species

Based on a cutoff value of 95% used for species delineation [61], the ANI values (> 99%) and the AAI values (>98%) observed between any two pairs of *Casuarina* isolates indicate that all *Casuarina* isolates belong to the same species, *Frankia casuarinae*, and are distinct from the closely related cluster Ia isolate *Frankia alni* strain ACN14a. The concatenated phylogenetic affiliation of 394 maximum-parsimony trees based on amino acid sequences also reveals that *Casuarina* isolates group together and are distinct from the closely related cluster Ia isolate strain ACN14a. The two highly salt-tolerant strains (Allo2 and CcI6) were the closest two strain having the lowest genome- to- genome distance, the highest ANI, and AAI. It is tempting to suggest that the salt tolerance mechanism of the two strains is shared. This idea was confirmed by the proteomic analysis of the two strains under salt stress which revealed a similar pattern of differentially expressed proteins. A shared salt-tolerance mechanism with a common origin for the two strains is further supported by pan-genome analysis of the *Casuarina* isolates which revealed hundreds of single copy genes that are exclusively shared between the two salt-tolerant strains. Transcriptome analysis revealed some of these tolerant strain-specific genes are responsive to salt and osmotic stress.

### Differences between RNA-seq and proteome results

After identifying genetic differences between the salt-tolerant and the salt-sensitive strains, we proceeded with transcriptomics and proteomics to determine if the genetic difference includes genes that are responsive to salt and osmotic stress. Because of factors such as half-lives and post transcription machinery, the correlation between mRNA and protein expressions can be low. Therefore, joint analysis of transcriptomic and proteomic data can provide useful insight that is otherwise impossible to obtain from individual analysis of mRNA or protein expressions [87]. Our results suggest that both transcriptional and post-transcriptional controls are involved in the regulation of genes under hyper-osmotic stress in strain CcI6.

### Altered expression of regulatory proteins

Alternative sigma factors and transcription regulators are involved in controlling expression of stress-responsive genes and help to create flexibility in the adaptation of cells to environmental stress [88]. Several genes encoding transcriptional factors from the GntR, TetR, LysR, and Crp/Fnr families were up-regulated under salt stress. Similarly, several genes encoding transcriptional regulators from the Crp/Fnr and LuxR families were up-regulated under osmotic challenge. Proteomics analysis did not reveal any transcriptional factors, probably because they are low abundance proteins that fall below the detection range. Under salt stress, only one sigma factor (CcI6_RS19210), which has a SigF domain, was up-regulated in *Frankia* sp. strain CcI6. A similar pattern of up-regulation under salt stress was also found in strains CcI3 and CeD (unpublished data). The sigF regulon in *Mycobacterium smegmatis* mediates stationary phase adaptation and general stress response [89]. *Mycobacterium smegmatis* SigF was suggested to regulate the biosynthesis of the osmoprotectant trehalose and an uptake system for osmoregulatory compounds. Our transcriptome results showed an up-regulation of trehalose synthase under salt stress. SigF has also been implicated in the direct control of gene expression for regulatory proteins SigH3, PhoP, WhiB1, and WhiB4. Under salt stress, only one sigma factor, an ECF sigma factor (CCI6_RS15595), was down-regulated under salt stress in *Frankia* strain CcI6. This result is in contrast with the reported auto-upregulation of ECF sigma factors in response to extracytoplasmic stress conditions, including salt stress [90]. ECF sigma factors recognize promoter elements with an ‘AAC’ motif in the −35 region and are usually co-transcribed with a transmembrane anti-sigma factor with an extracytoplasmic sensory domain and an intracellular inhibitory domain. In the strain CcI6 genome, the down-regulated ECF sigma factor lies upstream of a mycothiol system anti-sigma-R factor, suggesting that the ECF sigma factor is a homologue of sigma R. In *Streptomyces coelicolor* A3(2), sigma R is regulated by the cognate anti-sigma-R factor (RsrA), which loses affinity for sigma R following oxidative stress that introduces intramolecular disulphide bond formation in RsrA [91]. ECF sigma factors are regulated at the transcriptional, translational, and post-translational levels [90]. The transcriptional control of ECF factors can involve a hierarchical regulatory cascade of sigma factors. The most important regulation of ECF sigma factors involves the reversible binding of the sigma factor to an anti-sigma factor, holding it in an inactive complex as long as the cognate environmental stimulus is absent. Changes in environmental conditions sensed by the anti-sigma factor lead to release of the sigma factor, which will subsequently bind to the RNA polymerase core enzyme and initiate transcription [92]. Analysis of the proteome data, but not the transcriptome, showed that a σ^32^ factor involved in the cytoplasmic heat shock response is up-regulated under salt stress. This agrees with the result from the transcriptome analysis where the small heat-shock protein Hsp20 was up-regulated under salt stress.

### Potential mechanisms of salt tolerance

All *Casuarina* isolates had the same set of classical genes involved in salt and osmotic stress tolerance, suggesting the observed difference in tolerance was due to previously unknown or less characterized mechanisms. All *Casuarina* isolates lacked the BCCT family transporters, which are present in the closely related strain ACN14a, and lacked the ability to synthesize or acquire glycine betaine (or the precursor choline) from the environment. Strains CcI6 and Allo2 manifest high salt tolerance in a minimal growth medium confirming the idea that the ability to acquire glycine betaine from the environment is not a key factor in the salt tolerance of *Casuarina* isolates.

The majority of the hundreds of tolerant-strain-specific genes code for hypothetical proteins, suggesting novel mechanisms responsible for the tolerance. The remaining tolerant-strain-specific genes include those involved in replication, recombination and repair; and cell wall/membrane biogenesis. This would indicate that the ability to maintain the integrity of the genetic material, the replication process, and the cell envelope are all important for salt tolerance. The presence of unique genes in the salt-tolerant strains that are involved in cell wall and membrane biogenesis suggests that pre-existing differences in membrane/cell wall composition and structure might be contributing factors to the observed difference in salt tolerance levels between salt-sensitive and salt-tolerant strains.

Among the tolerant-strain-specific genes, only a small fraction was responsive to both salt and osmotic stress. The majority of those genes encoded hypothetical proteins, suggesting that novel mechanisms are responsible for the observed difference in salt-tolerance between strain CcI3 and the two salt-tolerant strains (CcI6 and Allo2). A zinc peptidase gene was among the salt-tolerant strain-specific genes that were responsive to salt stress. Transcriptome analysis of salt-tolerant and salt-sensitive varieties of rice had a zinc peptidase gene as one of the 50 top responsive genes [74]. The zinc peptidase gene up-regulated in strain CcI6 under salt stress has a DNA binding domain, suggesting it might play a role in response regulation. Salt stress and the accompanying oxidative stress leads to structural changes that compromise the function of proteins. Misfolded and aggregated proteins are degraded by proteases. Proteases are increasingly being associated with salt tolerance and sensitivity to abiotic stress [93]. None of the gene products unique to the salt-tolerant strains were identified from our proteomics analysis probably because they were present in amounts below the detection threshold.

From transcriptome analysis of strain CcI6, hundreds of genes were differentially expressed under salt and osmotic stress conditions. There was a clear overlap between salt and osmotic stress responses, but most of the responses were condition-specific. This result partly explains why there is a huge disparity between the salt and osmotic stress tolerance levels among the *Casuarina* isolates. The mechanism by which each functional category contributes towards salt-tolerance is discussed in the following paragraphs.

### Alterations of the cell envelope

The composition of the cell envelope plays an important role in osmo-adaptation [94]. Cell envelope-related changes triggered by salt stress include alterations in the structure and composition of the peptidoglycan layer [95], and changes in membrane and/or periplasmic protein composition, lipid composition, periplasmic glucan levels, and capsular polysaccharide biosynthesis [96]. These modifications to the cell wall under salt stress create a diffusion barrier to reduce the influx of inorganic ions [97]. Salt-stress induced alterations of the peptidoglycan layer involve several enzymes including glycolyl transferases, polysaccharide deacetylases and sugar epimerases. Our transcriptome data showed that several glycosyl transferases and nucleocide diphosphate sugar epimerases involved in cell wall/membrane biogenesis were up-regulated under both salt and osmotic stress conditions indicating that the cell envelope was being altered. However, different glycosyl transferases and nucleocide diphosphate sugar epimerases were up-regulated under each condition suggesting that these cell envelope changes were different under salt or osmotic stress.

### Changes in membrane fluidity

Regulation of membrane fluidity in response to osmotic stress is also an important aspect of cell envelope remodeling during salt stress [98]. Regulation of membrane fluidity mainly involves changes in the fatty acid composition of the membrane by varying the length of acyl chains, number of double bonds or branching of acyl chains by methyl groups [99]. Our transcriptome results show an up-regulation of ACP desaturases (CCI6_RS10965, CCI6_RS10965) genes only under salt stress, but not under osmotic stress. ACP desaturases catalyze the conversion of saturated fatty acids into unsaturated fatty acids by the introduction of at least one double bond. This result would indicate that the membrane has elevated levels of unsaturated fatty acids indicating an increased fluidity.

Salt stress also caused the up-regulation of *ubiE* (*CCI6_RS17660*) encoding a multispecies ubiquinone biosynthesis protein. In *E. coli,* ubiquinone accumulation increases salt tolerance through mechanical stabilization of the membrane [77]. Taken together, these results suggest that *Frankia* membrane fluidity was altered by salt stress.

### Compatible solutes

The accumulation of compatible solutes is one of the major physiological coping mechanisms employed by cells exposed to a hyper osmotic pressure. Among the commonly used compatible solutes are the sugar trehalose and various amino acids [30]. Genes coding for enzymes involved in trehalose synthesis were up-regulated under salt stress while genes coding for enzymes involved in the synthesis of threonine and glutamate were up-regulated under osmotic stress. Trehalose serves as a universal stress molecule and plays a role in the cellular adaptation to high osmolarity, heat, oxidation, desiccation and freezing [100]. The role of osmolytes transcends maintaining cell turgor by increasing intracellular osmolality. Molecular dynamics studies have demonstrated an interaction between the osmolyte trehalose and the membrane lipid head groups, although the observed resistance of membranes to strong osmotic stress could not fully be ascribed to the interaction [101]. The increased expression of the trehalose synthase gene only under chronic salt stress and the increase in expression of the threonine and glutamate synthase genes only under osmotic stress (induced by sucrose) suggests that the preferred protective osmolyte depends on the external solute causing the stress.

## Conclusions

Comparative genomics, transcriptome, and proteome analyses of *Frankia* strains isolated from *Casuarina* trees revealed that salt stress response involves differential expression of a myriad of genes from various functional categories. Most of the genes specific to the salt-tolerant strains coded for hypothetical proteins and many salt-stress responsive genes from the transcriptome and proteome profiles coded for hypothetical proteins. This line of evidence suggests a role for these hypothetical proteins in salt-stress response and may represent a previously uncharacterized novel mechanism(s) for salt tolerance. The development of genetic tools for the mutational analysis of some of the candidate genes coupled with biochemical and physiological analysis could yield insights on these novel mechanisms of salt tolerance.

## Additional files

Additional file 1: Table S1. List of primers used for qRT-PCR validation of RNA-seq data. (DOCX 12 kb)

Additional file 2: The effect of salt stress on the vesicle formation and nitrogenase activity by salt-tolerant and salt-sensitive *Casuarina isolates*. Cultures were grown under nitrogen-deficient conditions with various degrees of salt or osmotic stress. Panel (A) shows vesicle production. Panel (B) show nitrogenase activity expressed on a per-vesicle basis. (PPTX 64 kb)

Additional file 3: List of 153 single-copy genes shared between *Frankia* sp. strain CcI6 and Allo2, but are absent in *Frankia* sp. strain CcI3 and CeD, as determined by the modified lerat program. List of 132 genes present in *Frankia* sp. strain Allo2 and CcI6, but are absent in other Casuarina associated *Frankia* sp. strains (CcI3, CeD, Thr, and BMG5.23), as determined by OrthoVenn. (XLSX 13 kb)

Additional file 4: *Frankia* sp. strain CcI6 genes differentially expressed under salt and osmotic stress based on RNAseq analysis. (XLSX 87 kb)

Additional file 5: Two-dimensional polyacrylamide gel electrophoresis (PAGE) analysis of *Frankia* sp. strain Allo2 under control (no stress) conditions (A), 200 mM NaCl (B), and 200 mM sucrose (C). Red arrows indicate that proteins are up-regulated relative to the control, while yellow arrows indicate down regulated proteins relative to the control. The corresponding number spots were in-gel digested with trypsin and analyzed by liquid chromatography-mass spectrometry (LC-MS) and LC-MS/MS for protein identification. (PPTX 653 kb)

Additional file 6: Table S3. *Frankia* sp. strain Allo2 proteins differentially expressed under stress conditions. The identified proteins were classified by COG functional categories. Up-regulated proteins are shown by the upward pointing arrow (↑) whereas down-regulated proteins are shown by the downward pointing arrow (↓). No change (N/C) indicates that a spot was not picked for that particular condition because it showed similar intensity as the control. (DOCX 15 kb)

## Abbreviations

AAI: Average amino acid identity
ACN: Acetonitrile
ANI: Average nucleotide identity
CDS: Coding DNA sequence
CID: Low-energy collision-induced dissociation
COG: Cluster of Orthologous Groups of protein
DDH: DNA-DNA hybridization
Ddl: D-alanine ligase
DTT: Dithiothreitol
GGP: Geranylgeranyl pyrophosphate
GGPS: Geranylgeranyl pyrophosphate synthase
IAA: Iodoacetamide
IEF: Isoelectric focusing
IPG: Immobiline pH gradient
LC-MS: Liquid chromatography – mass spectrometry
MIC: Minimum inhibitory concentration
MTC: Maximum tolerable concentration
NAGGN: N-acetylglutaminylglutamine amide
OtsA: Trehalose-6-phosphate synthase
OtsB: Trehalose-6-phosphate phosphatase
qRT-PCR: Quantitative reverse transcription PCR
RIN: RNA integrity number
ROS: Reactive oxygen species
SDS: Sodium dodecyl sulfate
TCA: Trichloroacetic acid
