# Supplementary material for: Genomic, transcriptomic, and proteomic approaches towards understanding the molecular mechanisms of salt tolerance in Frankia strains isolated from Casuarina trees
Source: BMC Genomics. 2017 Aug 18;18:633. doi: 10.1186/s12864-017-4056-0 (PMC5563000; doi:10.1186/s12864-017-4056-0)
Supplement: Supplementary file 1 — List of primers used for qRT-PCR validation of RNA-seq data. (DOCX 12 kb) [file 12864_2017_4056_MOESM1_ESM.docx]

**Supplemental table 1.** Primers used for qRT-PCR validation of RNAseq data

| **Locus tag** | **Forward primer** | **Reverse primer** |
| --- | --- | --- |
| CCI6_RS21730 | TGC ACT TCT ATC GGC AAC C | GAA GTA CTG CGA CAG GAA GAA G |
| CCI6_RS17915 | TAG AGT TCC GTC CAG GTC TT | GGA CCG CAC AAC AGT CTT TA |
| CCI6_RS19875 | ACA CTC AAC GCA CGA ATC A | GTG TTG ATG CGG GTT ATC ATT TC |
| CCI6_RS17580 | AAG AAC TGG CGA ATC CTC AC | CGG ATT GCT GTT CGT TGA TTT |
| CCI6_RS01600 | CGA CAT CAA GAT CGA CCA CTA C | TTG GAC TTT CCG CCG TTT |
| CCI6_RS18570 | CCG GCA CTT CAC CTT CAT | CCG GAA GTG CGC GAT AA |
| CCI6_RS06495 | CGT CGC AAC CTC TAC ATC TAC | CGG GAT GAA CTG GAT GAC AA |
| CCI6_RS02325 | CAA CGG GCA GGT GAT CTA TT | GAA TCC GTC AAC ACG CTC T |
| CCI6_RS12340 | GCA GAA CCA GCT CTT CCC | AAC GGC TGGAAC CAG AAC |
| CCI6_RS08505 | GGG TGA AGG GTG ATC CTT ATG | GTT GAT CAT GGA TGG CAG GTA |
| CCI6_RS19950 | ATA CGC TTC TGC TCG TGA AC | CCG GCA CGA TCT GTG TAA ATA |
