# Supplementary material for: Genomic, transcriptomic, and proteomic approaches towards understanding the molecular mechanisms of salt tolerance in Frankia strains isolated from Casuarina trees
Source: BMC Genomics. 2017 Aug 18;18:633. doi: 10.1186/s12864-017-4056-0 (PMC5563000; doi:10.1186/s12864-017-4056-0)
Supplement: Supplementary file 2 — The effect of salt stress on the vesicle formation and nitrogenase activity by salt-tolerant and salt-sensitive Casuarina isolates. Cultures were grown under nitrogen-deficient conditions with various degrees of salt or osmotic stress. Panel (A) shows vesicle production. Panel (B) show nitrogenase activity expressed on a per-vesicle basis. (PPTX 64 kb) [file 12864_2017_4056_MOESM2_ESM.pptx]

## Slide 1
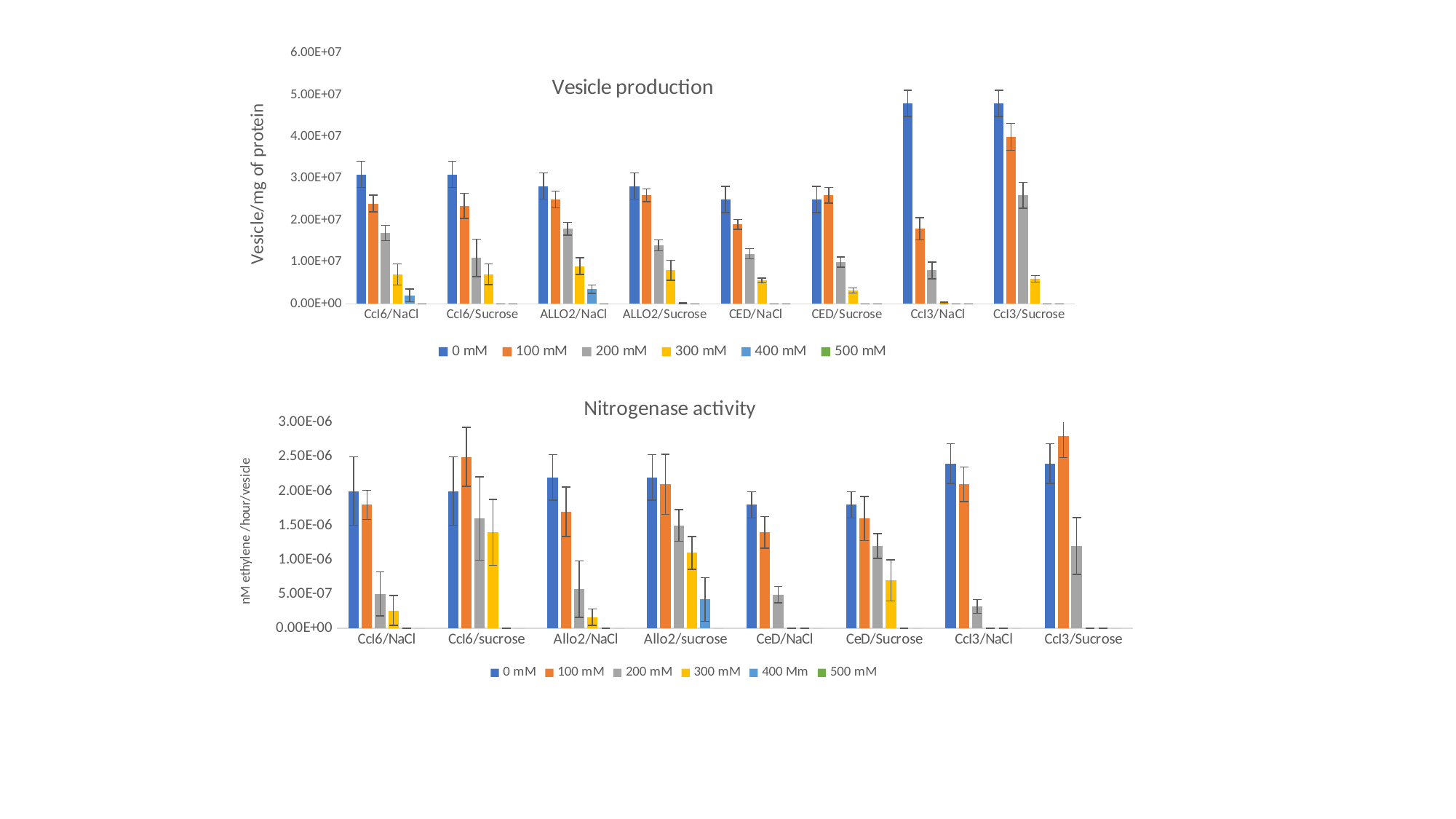

### Chart: Vesicle production
| Category | 0 mM | 100 mM | 200 mM | 300 mM | 400 mM | 500 mM |
|---|---|---|---|---|---|---|
| CcI6/NaCl | 31000000.0 | 24000000.0 | 17000000.0 | 7000000.0 | 2000000.0 | 0.0 |
| CcI6/Sucrose | 31000000.0 | 23500000.0 | 11000000.0 | 7100000.0 | 0.0 | 0.0 |
| ALLO2/NaCl | 28200000.0 | 25000000.0 | 18000000.0 | 9000000.0 | 3500000.0 | 0.0 |
| ALLO2/Sucrose | 28200000.0 | 26000000.0 | 14000000.0 | 8000000.0 | 100000.0 | 0.0 |
| CED/NaCl | 25000000.0 | 19000000.0 | 12000000.0 | 5600000.0 | 0.0 | 0.0 |
| CED/Sucrose | 25000000.0 | 26000000.0 | 10000000.0 | 3200000.0 | 0.0 | 0.0 |
| CcI3/NaCl | 48000000.0 | 18000000.0 | 8000000.0 | 200000.0 | 0.0 | 0.0 |
| CcI3/Sucrose | 48000000.0 | 40000000.0 | 26000000.0 | 6000000.0 | 0.0 | 0.0 |
### Chart: Nitrogenase activity
| Category | 0 mM | 100 mM | 200 mM | 300 mM | 400 Mm | 500 mM |
|---|---|---|---|---|---|---|
| CcI6/NaCl | 2e-06 | 1.8e-06 | 5e-07 | 2.6e-07 | 0.0 | 0.0 |
| CcI6/sucrose | 2e-06 | 2.5e-06 | 1.6e-06 | 1.4e-06 | 0.0 | 0.0 |
| Allo2/NaCl | 2.2e-06 | 1.7e-06 | 5.7e-07 | 1.6e-07 | 0.0 | 0.0 |
| Allo2/sucrose | 2.2e-06 | 2.1e-06 | 1.5e-06 | 1.1e-06 | 4.2e-07 | 0.0 |
| CeD/NaCl | 1.8e-06 | 1.4e-06 | 4.9e-07 | 0.0 | 0.0 | 0.0 |
| CeD/Sucrose | 1.8e-06 | 1.6e-06 | 1.2e-06 | 7e-07 | 0.0 | 0.0 |
| CcI3/NaCl | 2.4e-06 | 2.1e-06 | 3.2e-07 | 0.0 | 0.0 | 0.0 |
| CcI3/Sucrose | 2.4e-06 | 2.8e-06 | 1.2e-06 | 0.0 | 0.0 | 0.0 |
