# Supplementary figures and images for: Genomic, transcriptomic, and proteomic approaches towards understanding the molecular mechanisms of salt tolerance in Frankia strains isolated from Casuarina trees
Source: BMC Genomics. 2017 Aug 18;18:633. doi: 10.1186/s12864-017-4056-0 (PMC5563000; doi:10.1186/s12864-017-4056-0)

## Slide 1
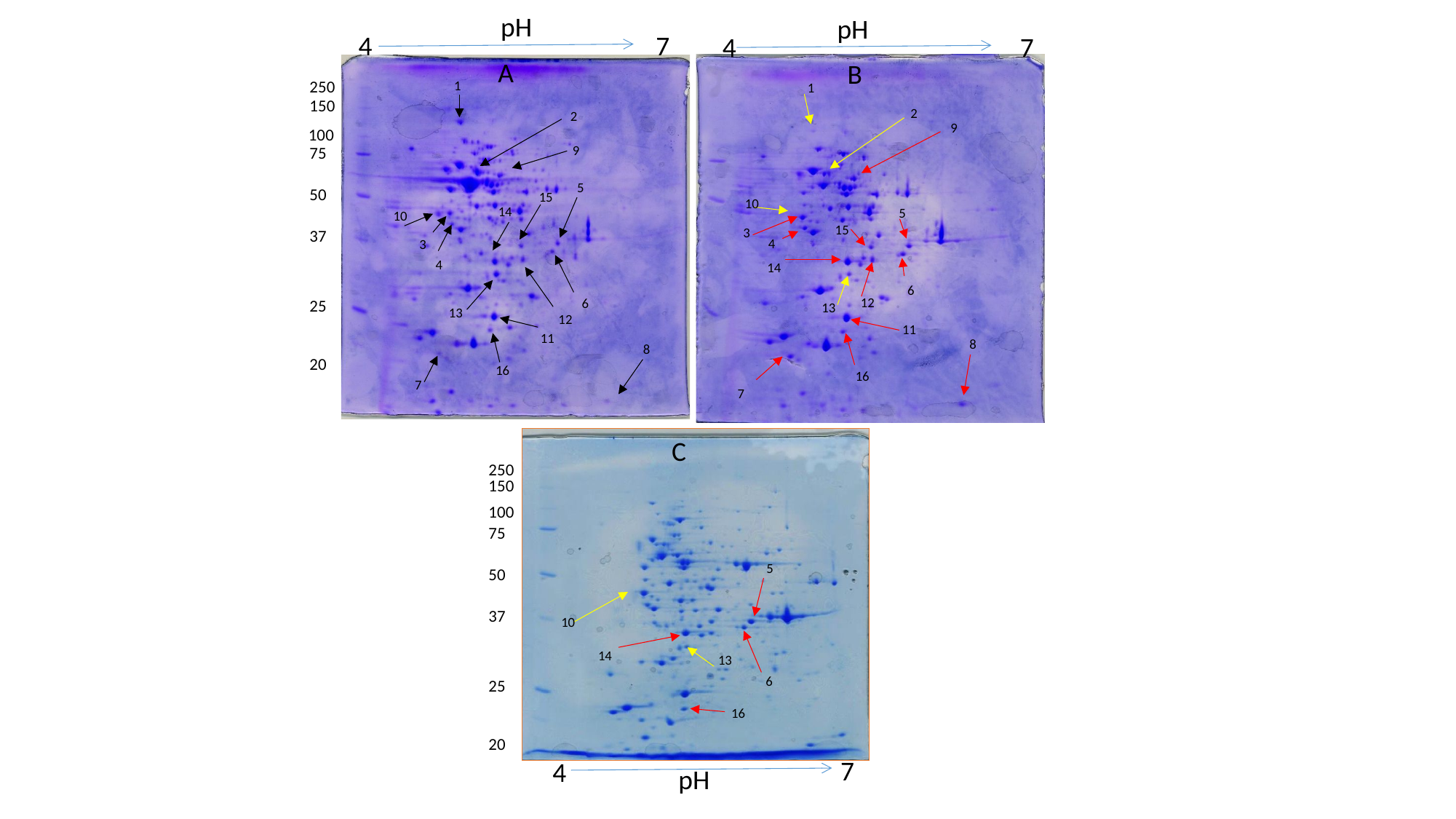

pH
pH
 4 7
 4 7
A
B
1
2
10
5
3
4
6
8
7
1
2
10
3
4
6
7
250
150
100
75
50
37
25
20
9
5
8
5
10
6
250
150
100
75
50
37
25
20
7
4
pH
15
14
15
14
12
13
13
12
11
11
14
13
16
16
9
C
16

Supplement: Supplementary file 5 — Two-dimensional polyacrylamide gel electrophoresis (PAGE) analysis of Frankia sp. strain Allo2 under control (no stress) conditions (A), 200 mM NaCl (B), and 200 mM sucrose (C). Red arrows indicate that proteins are up-regulated relative to the control, while yellow arrows indicate down regulated proteins relative to the control. The corresponding number spots were in-gel digested with trypsin and analyzed by liquid chromatography-mass spectrometry (LC-MS) and LC-MS/MS for protein identification. (PPTX 653 kb) [file 12864_2017_4056_MOESM5_ESM.pptx]
