# Supplementary material for: Genomic, transcriptomic, and proteomic approaches towards understanding the molecular mechanisms of salt tolerance in Frankia strains isolated from Casuarina trees
Source: BMC Genomics. 2017 Aug 18;18:633. doi: 10.1186/s12864-017-4056-0 (PMC5563000; doi:10.1186/s12864-017-4056-0)
Supplement: Supplementary file 6 — Frankia sp. strain Allo2 proteins differentially expressed under stress conditions. The identified proteins were classified by COG functional categories. Up-regulated proteins are shown by the upward pointing arrow (↑) whereas down-regulated proteins are shown by the downward pointing arrow (↓). No change (N/C) indicates that a spot was not picked for that particular condition because it showed similar intensity as the control. (DOCX 15 kb) [file 12864_2017_4056_MOESM6_ESM.docx]

**Supplemental table 3.** Frankia sp. strain Allo2 proteins differentially expressed under stress conditions. The identified proteins were classified by COG functional categories. Upregulated proteins are shown by the upward pointing arrow (↑) whereas downregulated proteins are shown by the downward pointing arrow (↓). No change (N/C) indicates that a spot was not picked for that particular condition because it showed similar intensity as the control.

| **SPOT #** | **Acc. No** | **Locus Tag** | **Protein Name** | **MW (Da)** | **PI** | **NaCl** | **Sucrose** |
| --- | --- | --- | --- | --- | --- | --- | --- |
| **[C] Energy production and Conversion** | | | | | | | |
| 1 | WP_035732921.1 | ALLO2_RS15565 | aconitate hydratase | 98737.30 | 4.80 | ↓ | N/C |
| 4 | WP_035729778.1 | ALLO2_RS03755 | malate dehydrogenase | 41530.50 | 4.80 | ↑ | N/C |
| 14 | WP_011437820.1 | ALLO2_RS10070 | malate dehydrogenase (NAD) | 34399.10 | 4.96 | ↑ | ↑ |
| 13 | WP_035734595.1 | ALLO2_RS22325 | electron transfer flavoprotein alpha subunit apoprotein | 32842.70 | 5.01 | ↓ | ↓ |
| **[E] Amino acid transport and metabolism** | | | | | | | |
| 13 | WP_035732776.1 | ALLO2_RS14775 | cysteine synthase (CysK) | 32443.60 | 4.95 | ↑ | ↑ |
| **[G] Carbohydrate transport and metabolism** | | | | | | | |
| 3 | WP_035729979.1 | ALLO2_RS04610 | enolase | 44751.80 | 4.60 | ↑ | N/C |
| 5 | WP_023841695.1 | ALLO2_RS05865 | glyceraldehyde-3-phosphate dehydrogenase | 35515.80 | 5.70 | ↑ | ↑ |
| 6 | WP_035729149.1 | ALLO2_RS01575 | fructose-bisphosphate aldolase | 36894.20 | 5.35 | ↑ | ↑ |
| **[H] Coenzyme metabolism** | | | | | | | |
| 12 | WP_035732888.1 | ALLO2_RS15275 | pyridoxal phosphate synthase yaaD subunit | 32577.80 | 5.34 | ↑ | N/C |
| **[k] Transcription** | | | |  |  |  |  |
| 10 | WP_011435063.1 | ALLO2_RS03340 | DNA-directed RNA polymerase subunit alpha | 37859.00 | 4.60 | ↓ | ↓ |
| **[I] Lipid transport and metabolism** | | | | | | | |
| 11 | WP_035732830.1 | ALLO2_RS14885 | short chain enoyl-CoA hydratase | 26841.10 | 4.98 | ↑ | N/C |
| [**M] Cell wall/membrane/envelop biogenesis** | | | | | | | |
| 15 | WP_023840985.1 | | UDP-glucose pyrophosphorylase | 34856.10 | 5.04 | ↑ | N/C |
| **[O] Post-translational modification, protein turnover, chaperone functions** | | | | | | | |
| 2 | WP_011435086.1 | ALLO2_RS03455 | molecular chaperone GroEL | 57446.70 | 4.90 | ↓ | N/C |
| 7 | WP_011435509.1 | ALLO2_RS18350 | glutathione peroxidase | 19567.10 | 4.70 | ↑ | N/C |
| 8 | WP_011438798.1 | ALLO2_RS01170 | peptidyl-prolyl cis-trans isomerase | 19104.60 | 6.10 | ↑ | N/C |
| 16 | WP_035730024.1 | ALLO2_RS05025 | ATP-dependent Clp protease proteolytic subunit ClpP | 23039.30 | 4.79 | ↑ | ↑ |
| **Not assigned to COG categories** | | | | | | | |
| 9 | WP_035733723.1 | ALLO2_RS18330 | carnitine O-acetyltransferase | 67431.10 | 5.20 | ↑ | N/C |
